# Supply of Methionine During Late-Pregnancy Alters Fecal Microbiota and Metabolome in Neonatal Dairy Calves Without Changes in Daily Feed Intake

**DOI:** 10.3389/fmicb.2019.02159

**Published:** 2019-09-19

**Authors:** Ahmed Elolimy, Abdulrahman Alharthi, Mohamed Zeineldin, Claudia Parys, Ariane Helmbrecht, Juan J. Loor

**Affiliations:** ^1^Mammalian NutriPhysioGenomics, Department of Animal Sciences, University of Illinois at Urbana–Champaign, Urbana, IL, United States; ^2^Department of Animal Sciences, University of Illinois at Urbana–Champaign, Urbana, IL, United States; ^3^Department of Animal Production, National Research Centre, Giza, Egypt; ^4^Carl R. Woese Institute for Genomic Biology, University of Illinois at Urbana–Champaign, Urbana, IL, United States; ^5^Department of Animal Medicine, College of Veterinary Medicine, Benha University, Toukh, Egypt; ^6^Evonik Nutrition & Care GmbH, Hanau-Wolfgang, Germany; ^7^Division of Nutritional Sciences, Illinois Informatics Institute, University of Illinois Urbana–Champaign, Urbana, IL, United States

**Keywords:** prenatal, methionine, cows, calves, preweaning, gut, microbiota, metabolomics

## Abstract

To our knowledge, most studies demonstrating the role of manipulating maternal nutrition on hindgut (i.e., large intestine) microbiota in the offspring have been performed in non-ruminants. Whether this phenomenon exists in cattle is largely unknown. Therefore, the objectives of the current study were to evaluate the impact of maternal post-ruminal supply of methionine during late-pregnancy in dairy cows on fecal microbiota and metabolome in neonatal calves, and their association with body development and growth performance during the preweaning period. To achieve this, heifer calves, i.e., neonatal female offspring, born to Holstein cows receiving either a control (CON) diet (*n* = 13) or CON plus rumen-protected methionine (MET; Evonik Nutrition & Care GmbH) during the last 28 days of pregnancy were used. Fecal samples from heifers were collected from birth until 6 weeks of age, i.e., the preweaning period. Fecal microbiota was analyzed with QIIME 2 whereas fecal metabolites were measured using an untargeted LC-MS approach. At birth, MET heifers had greater (*P* ≤ 0.05) BW, HH, and WH. During the preweaning period, no differences between groups were detected for starter intake (*P* = 0.77). However, MET heifers maintained greater (*P* ≤ 0.05) BW, HH and tended (*P* = 0.06) to have greater WH and average daily gain (ADG) (*P* = 0.10). Fecal microbiota and metabolome profiles through 42 days of age in MET heifers indicated greater capacity for hindgut production of endogenous antibiotics and enhanced hindgut functionality and health. Enhancing maternal post-ruminal supply of methionine during late-gestation in dairy cows has a positive effect on hindgut functionality and health in their offspring through alterations in the fecal microbiota and metabolome without affecting feed intake. Those alterations could limit pathogen colonization of the hindgut while providing essential nutrients to the neonate. Together, such responses contribute to the ability of young calves to achieve better rates of nutrient utilization for growth.

## Introduction

The hindgut microbiota contribute substantially to the regulation of host metabolism, immune response and other crucial physiological processes via the production of numerous bioactive metabolites such as volatile fatty acids (VFA), essential amino acids, vitamins and neurotransmitters (Thursby and Juge, 2017). These coordinated processes promote growth and development in dairy calves (Malmuthuge and Guan, 2017). Although it is well-established that the hindgut microbiota is crucial for the functionality of gastrointestinal tract in non-ruminants, whether it can be programmed during pregnancy or early life in ruminants remains largely unknown. The early hindgut microbiota can be determined, at least in part, through vertical transfer of maternal microorganisms *in utero* (Zhu L. et al., 2018). A growing number of studies across non-ruminant species highlight the central role of maternal diet during pregnancy on the programming of the microbiota in the offspring (Paul et al., 2016; Zhang et al., 2019). For example, in humans, Chu et al. (2016) reported that maternal high-fat diet intake (43.1% fat content in the diet) during late-pregnancy (last 12 weeks of gestation) induced distinct changes in neonatal hindgut microbiota that persisted until 6 weeks of age. *Enterococcus* were enriched and *Bacteroides* reduced in neonates exposed to maternal high-fat intake compared with controls (24.4% fat content in the diet) (Chu et al., 2016). In another study with pigs, Cheng et al. (2018) detected that soluble fiber supply throughout pregnancy induced marked changes in hindgut microbiota composition of 2-week-old piglets, including increased *Lactobacillus*, *Bacteroides* and *Roseburia*. Those profiles were positively correlated with better hindgut development including an increase in energy extraction from polysaccharides and a reduction in intestinal permeability and inflammation, all of which led to greater growth performance in piglest (Cheng et al., 2018).

Ensuring a proper post-ruminal supply of methionine to dairy cows during the periparturient period has garnered interest in recent years due to beneficial effects of this amino acid in alleviating unfavorable health consequences of negative protein and energy balance around parturition (Osorio et al., 2016; Batistel et al., 2017b; Zhou et al., 2018). In addition, other studies have underscored beneficial effects of maternal supply of methionine to neonatal calves, including greater body size (Alharthi et al., 2018), faster maturation of hepatic metabolic pathways (Jacometo et al., 2017) and better innate immune function (Jacometo et al., 2018) during the preweaning period. Besides these physiologic effects, enhanced maternal methionine supply during late-pregnancy induced distinctive changes in composition of the hindgut microbiota and plasma metabolome in piglets (Azad et al., 2018). Those changes were proposed to occur via microbial uptake and metabolism of methionine in the hindgut (Neis et al., 2015). Enhanced maternal supply of choline, betaine, folate, and vitamin B_12_ during pregnancy in mice induced persistent changes in hindgut microbiota of the offspring during the preweaning period (Schaible et al., 2011). Whether maternal methionine supply, a key methyl donor, during pregnancy in dairy cows affects the hindgut microbiota of calves is unknown.

To date, most published studies have focused on the variation of phylogenetic architecture in the hindgut microbiota, while few studies investigated hindgut metabolites alongside microbial composition. Thus, a number of unanswered questions remain especially regarding potential changes in hindgut metabolome driven by manipulation of the microbiota. Profiling the fecal metabolome might provide valuable information on the potential adaptive responses of the hindgut microbiota in the calf to changes in maternal diet during pregnancy. Furthermore, these data could help determine associations between hindgut function and efficiency of nutrient use for growth early in life.

We hypothesized that enhanced post-ruminal supply of methionine in late-pregnancy is associated with development of a unique hindgut microbiota and metabolome in neonatal calves that helps explain in part better growth performance during early life. To address this hypothesis, we studied changes in fecal microbiota and metabolome, representing hindgut microbial communities and metabolites (Li Z. et al., 2018), in neonatal female calves born to cows fed a control diet or control plus rumen-protected methionine during the last 28 days of pregnancy (Batistel et al., 2017a). Fecal samples were harvested from birth until 6 weeks of age, i.e., during the preweaning period. These changes in hindgut microbiota-metabolome were evaluated in the context of growth performance of the same calves.

## Materials and Methods

The research protocol was reviewed and approved by the Institutional Animal Care and Use Committee of the University of Illinois (protocol no. 14270).

### Maternal Treatments

Details of maternal treatments have been described previously (Batistel et al., 2017a). Briefly, 60 multiparous pregnant Holstein cows received a common early dry period diet, i.e., far-off diet, from −45 to −29 days relative to parturition. All cows received a low-energy and high-straw far-off diet containing 1.33 Mcal/kg of dry matter (DM) and 13.9% crude protein (CP) with no added methionine. Cows were individually fed using the Calan Broadbent Feeding System (American Calan Inc., Northwood, NH, United States). At −28 days relative to parturition, cows were randomly assigned to receive either a basal control (CON) close-up diet (*n* = 30; 1.47 Mcal/kg DM and 15.3% CP) with no added methionine or CON plus methionine added in the form of ethyl-cellulose rumen-protected methionine (MET, *n* = 30; Mepron^®^, Evonik Nutrition & Care GmbH, Germany). The ingredient and nutrient composition of the “far-off” and “close-up” diet are reported in Supplementary Table S1 and Supplementary Table S2. All diets were formulated to meet cow predicted requirements according to NRC (2001). Mepron is a commercial source of rumen-protected methionine in the form of small beads containing a minimum of 85% methionine, including an equimolar mixture of D-methionine and L-methionine isomers. Mepron beads resist microbial degradation in the rumen due to an ethyl-cellulose film coating the methionine core, resulting in ruminal bypass value of 80% (Overton et al., 1996). Mepron digestibility coefficient in the intestine is 90% (Schwab, 1995), therefore, every 10 g of Mepron provides the cow with 6.1 g metabolizable methionine. Mepron was top-dressed once daily on the total mixed ration (TMR) during the close-up period from −28 days to calving date at a rate of 0.09% of previous day dry matter intake (DMI). This rate was based on studies demonstrating beneficial effects on production performance and health during the prepartum period (Osorio et al., 2013; Zhou et al., 2016). After calving, colostrum volume was recorded.

### Enrolment Criteria and Management of Neonatal Heifers

After parturition, neonatal calves were separated from their dams. Heifer calves were kept in the experiment if they fulfilled all the following criteria described previously by Jacometo et al. (2016): (1) single heifer calf; (2) heifer calf birth weight >36 kg; (3) calving difficulty score <3; and (4) dam first colostrum volume >3.8 L. A subset of heifer calves born to cows receiving CON (*n* = 13) or MET (*n* = 13) diets were selected randomly for the current study. All heifer calves were managed in the same fashion during the first 6 weeks of life. At birth, the navel was disinfected with 7% tincture of iodine solution (First Priority Inc., Elgin, IL, United States), and neonatal heifers were vaccinated with TSV II (Pfizer Inc., New York, NY, United States) via nostril application. Calves received 3.8 L of first-milking colostrum collected from their dams within 6 h after birth. They were housed in individual outdoor hutches bedded with straw and fed twice daily (morning and afternoon) with a milk replacer (Advance Excelerate, Milk Specialties, Carpentersville, IL, United States; 28.5% CP, 15% fat) until 35 days of age. The nutrient composition and amino acid profiles of the milk replacer are reported in Table 1. At d 36, neonatal heifers were switched to once-daily milk replacer feeding at the morning until weaning (42 days of age). Heifer calves received 4.54 kg/day of milk replacer mix (0.59 kg of milk replacer in 3.95 L of water) from 1 to 10 days of age, 5.90 kg/day (0.77 kg of milk replacer in 5.13 L of water) from 11 to 20 days of age, 7.26 kg/day (0.94 kg of milk replacer in 6.32 L of water) from 21 to 35 days of age and 3.63 kg/day (0.47 kg of milk replacer in 3.16 L of water) from 36 to 42 days of age. From day 1 until day 42 of life, neonatal heifers had *ad libitum* access to starter grain mix [Ampli-Calf Starter 20^®^; 19.9% CP and 13.5% neutral detergent fiber (NDF), Purina Animal Nutrition, Shoreview, MN, United States) fed in the morning. Nutrient composition and amino acid profiles of the starter grain mix are reported in Table 1. Starter intake was recorded daily until 42 days of age. Body measurements including body weight (BW), hip height (HH) and width (HW), wither height (WH) and body length (BL) were measured weekly at day 0 (i.e., at birth before colostrum feeding), 7, 14, 21, 28, 35, and 42. Weekly BW gain was calculated as final BW at the end of the week minus initial BW at the beginning of the week divided by total days per week (i.e., 7 days) whereas cumulative BW gain was calculated as BW at 42 days of age minus initial BW at birth. Overall feed conversion ratio (FCR) was calculated as cumulative BW gain divided by cumulative starter DMI from birth to 42 days of age. All heifer calves remained clinically healthy during the study.

**TABLE 1:** Nutrient composition and amino acid profiles (mean ± standard deviation) of milk replacer (Advance Excelerate, Milk Specialties, Carpentersville, IL, United States) and starter grain (Ampli-Calf Starter 20; Purina Animal Nutrition, Shoreview, MN, United States) fed during preweaning period to heifer calves born to cows offered a control diet (CON, *n* = 13) or CON supplemented with ethyl-cellulose rumen-protected methionine (MET, *n* = 13; Mepron^®^ at 0.09% of diet DM; Evonik Nutrition & Care GmbH, Germany) during the last 28 days of pregnancy.

**Item**	**Milk replacer**	**Starter grain mix**
DM, g/kg	946 ± 11	908 ± 27
CP, g/kg	272 ± 44	209 ± 23
**Essential amino acids, % of DM**		
Arginine	0.84 ± 0.07	1.39 ± 0.03
Histidine	0.59 ± 0.02	0.55 ± 0.01
Isoleucine	1.60 ± 0.06	0.82 ± 0.01
Leucine	2.90 ± 0.07	1.52 ± 0.03
Lysine	2.30 ± 0.12	1.07 ± 0.02
Methionine	0.53 ± 0.02	0.29 ± 0.01
Phenylalanine	1.01 ± 0.02	0.98 ± 0.01
Threonine	1.83 ± 0.04	0.77 ± 0.02
Valine	1.59 ± 0.03	0.97 ± 0.01
**Non-essential amino acids, % of DM**		
Aspartate	2.89 ± 0.07	2.00 ± 0.05
Alanine	1.36 ± 0.03	0.97 ± 0.02
Cysteine	0.64 ± 0.02	0.34 ± 0.01
Glutamate	4.68 ± 0.15	1.07 ± 0.02
Glycine	0.60 ± 0.03	0.98 ± 0.01
Proline	1.64 ± 0.04	1.14 ± 0.02
Serine	1.47 ± 0.05	0.99 ± 0.03

### Fecal Sampling and Storage

Fecal samples were obtained at birth in duplicate from each heifer before colostrum feeding using sterile double sheathed equine uterine culture swabs (EquiVet, Kruuse, Denmark) inserted 10 cm into the rectum. The swab was only exposed to the rectum. For samples at day 14, 28, and 42, heifers were rectally finger-stimulated with a sterile-gloved hand to facilitate the collection of fresh fecal material that was subsequently placed in a sterile Whirl-Pak^®^ bag (Nasco, Fort Atkinson, WI, United States). Fecal swabs and bags were immediately flash frozen in liquid nitrogen and stored at −80°C for microbiota and metabolome analyses.

### Fecal DNA Extraction, 16S rRNA Gene Amplification and Sequencing

Total DNA was extracted from fecal samples (single fecal swab or 100 mg feces from fecal bags) using the DNeasy PowerSoil kit (Qiagen, Valencia, CA, United States) in accordance with manufacturer’s instructions. To track any contamination during the DNA extraction, 3 no-template negative controls (i.e., samples without biological material) were processed to assess the presence of microbial contamination in the swabs and the extraction reagents. The negative controls were run through the entire workflow alongside samples for quality control. Total DNA concentration and integrity were evaluated using a NanoDrop spectrophotometer (ND 1000, NanoDrop Technologies, Inc., Wilmington, DE, United States) and via visualization in a 2% (wt/v) agarose gel electrophoresis (Sigma-Aldrich, Saint Louis, MO, United States) with SYBR Safe DNA Gel Stain (Invitrogen, Grand Island, NY, United States). The extracted DNA was immediately stored at −80°C for further analysis. All DNA samples were quantified on a Qubit fluorometer (Life technologies, Grand Island, NY, United States) using the High Sensitivity DNA Kit (Roche, Indianapolis, IN, United States) and 20x Access Array loading reagent. Total DNA was subjected to Fluidigm Access Array Amplification (Fluidigm Corporation, South San Francisco, CA, United States) for DNA amplification. The 16S rDNA V3-V4 region of the prokaryotic ribosomal RNA gene was amplified by PCR (95°C for 2 min, followed by 27 cycles at 98°C for 10 s, 62°C for 30 s, and 68°C for 30 s and a final extension at 68°C for 10 min) using primers 341F: CCTACGGGNGGCWGCAG; 806R: GGACTACHVGGGTATCTAAT, where CS1 forward and CS2 reverse tags with unique eight-base sequence barcode were added to each sample according to the Fluidigm protocol instructions (Fluidigm Corporation, South San Francisco, CA, United States). The PCR reactions were performed in triplicate 50 μL mixtures each containing 5 μL of 10 × KOD Buffer, 5 μL of 2.5 mM dNTPs, 1.5 μL of each primer (5 μM), 1 μL of KOD Polymerase, and 100 ng of template DNA (He et al., 2019).

The final PCR product was quantified on a Qubit fluorometer (Life technologies, Grand Island, NY, United States) and the quality of amplicon regions assessed using a Fragment Analyzer (Advanced Analytics, Ames, IA, United States) to confirm amplicon regions and sizes. DNA samples were pooled in equal amounts according to concentration. Pooled samples were then size-selected on a 2% agarose gel (Life Technologies, Grand Island, NY, United States) and extracted using the Qiagen gel purification kit (Qiagen, Valencia, CA, United States). Cleaned size-selected pooled products were run on an Agilent Bioanalyzer to confirm appropriate profile and average size. The final pooled Fluidigm libraries were transferred to the DNA Services lab at the W. M. Keck Center for Comparative and Functional Genomics at the University of Illinois at Urbana-Champaign, Illinois, United States for Illumina sequencing. The Illumina MiSeq V2 platform (Illumina, San Diego, CA, United States) was used to sequence the V3-V4 region of the 16S rRNA gene according to Illumina instructions. The libraries were sequenced from both ends of the molecules to a total read length of 300 nt from each end according to Illumina instructions (Zeineldin et al., 2018). Data quality filters on the raw microbiota sequences were applied with Illumina software. Any reads detected in the negative control were filtered out of the data analysis.

### Analysis of Amplicon Sequencing Data

High quality 16S rRNA amplicon sequences were analyzed with the open source Quantitative Insights into Microbial Ecology (QIIME) 2.0 (Bolyen et al., 2019). Reads were de-noised into amplicon sequence variants (ASVs) using the DADA2 pipeline as implemented in QIIME 2.0 (Callahan et al., 2016). Taxonomic classification of sequences was assigned to ASVs using the feature classifier against the Greengenes 16S rRNA gene database version13.8 (McDonald et al., 2012). Bacterial alpha diversity indices, including Shannon, Chao1 and observed species indices per sample were calculated using QIIME 2.0. We visualized differences in beta-diversity with non-metric multidimensional scaling (NMDS) plots, which were constructed using MicrobiotaAnalyst (Dhariwal et al., 2017). Bar/line taxonomy plots depicting the most-prevalent fecal taxa, averaged >0.01% of the relative abundance across all samples, were generated in JMP 13.2 (SAS Institute Inc., Cary, NC, United States). A cladogram of LEfSe analysis for overrepresented microbes between CON and MET groups was obtained using the Galaxy workflow framework (Segata et al., 2011). The PICRUSt 1.1.2 (Phylogenetic Investigation of Communities by Reconstruction of Unobserved States) pipeline (Langille et al., 2013) and STAMP 2.1.3 (Parks et al., 2014) were used to investigate and illustrate alterations in microbial functions of fecal the microbiota in response to maternal post-ruminal supply of methionine.

### Fecal Metabolite Extraction and LC-MS Analysis

Free metabolites were extracted as described by Yu et al. (2017) with modifications. Fecal samples (single fecal swab or 100 mg feces from fecal bags) were dissolved in 1 mL ice cold purified water prepared using on a Milli-Q water purification system (Millipore Corp, Bedford, MA, United States). The mixture was vortexed and centrifuged at 10,000 × *g* for 15 min at 4°C. Supernatant was collected and kept on ice, whereas the remaining fecal pellet was further extracted by adding 1 mL ice cold LC-MS grade methanol (Sigma-Aldrich, Steinheim, Germany). The mixture was vortexed and centrifuged at 10,000 × *g* for 15 min at 4°C. Supernatant was collected and kept on ice. Both fecal supernatants were combined and centrifuged at 10,000 × *g* for 15 min at 4°C. The resulting supernatant was collected and stored at −80 °C until LC–MS analysis.

Samples were analyzed with the Q-Exactive MS system (Thermo. Bremen, Germany) at the Metabolomics Laboratory of Roy J. Carver Biotechnology Center, University of Illinois at Urbana-Champaign, United States. Software Xcalibur 4.1.31.9 was used for data acquisition. The Dionex Ultimate 3000 series HPLC system (Thermo, Germering, Germany) used had a degasser, an autosampler and a binary pump. The LC separation was performed on a Phenomenex Kinetex C18 column (4.6 × 100 mm, 2.6 μm) with mobile phase A (H_2_O with 0.1% formic acid) and mobile phase B (acetonitrile with 0.1% formic acid). The flow rate was 0.25 mL/min. The linear gradient was as follows: 0–3 min, 100% A; 20–30 min, 0% A; 31–36 min, 100% A. The autosampler was set to 15°C and injection volume was 20 μL. Mass spectra were acquired under both positive (sheath gas flow rate: 45; aux gas flow rate: 11; sweep gas flow rate: 2; spray voltage: 3.5 kV; capillary temp: 250°C; Aux gas heater temp: 415°C) and negative electrospray ionization (sheath gas flow rate: 45; aux gas flow rate: 11; sweep gas flow rate: 2; spray voltage: −2.5 kV; capillary temp: 250°C; Aux gas heater temp: 415°C). The full scan mass spectrum resolution was set to 70,000 with scan range of m/z 67 ∼ m/z 1,000, and AGC target was 1E6 with a maximum injection time of 200 ms. The 4-Chloro-DL-phenylalanine was spiked into the sample as the internal standard. LC-MS data were further analyzed with Thermo Compound Discoverer software (v. 2.1 SP1) for chromatographic alignment and compound/feature identification/quantitation. The workflow is Untargeted Metabolomics with Statistics Detect Unknowns with ID Using Online Databases. The following settings were used in Select Spectra: minimum precursor mass (65 Da) and maximum precursor mass (5,000 Da); in Align Retention Time: Maximum shift (1 min) and Mass tolerance (5 ppm); in Detect unknown compounds: Mass tolerance (5 ppm), Intensity tolerance (30 %), S/N (3), and Minimum peak intensity (1000000).

### Metabolomics Data Processing

Data visualization and statistical analyses for fecal metabolome data were performed with MetaboAnalyst 4.0 (Chong et al., 2018). The raw data were checked for data integrity and normalized by sum and autoscaling in order to enhance the performance for downstream statistical analysis (Khan et al., 2018). Multivariate analysis was performed via the supervised partial least squares discriminant analysis (PLS-DA). This allowed visualization metabolic profile dissimilarities between CON and MET groups, identification of important metabolites separating the two groups and trends in upregulation or downregulation in MET group (Meloni et al., 2018). Metabolites most strongly influencing discrimination between the two groups were selected according to their importance in differentiating the metabolic profile between maternal dietary groups based on the following criteria: variable importance in the projection (VIP) score > 1.0 and |*p*-(corr)| ≥ 0.5 with 95% jack-knifed confidence intervals (Yan et al., 2017). Based on the identification level 3 of Metabolomics Standards Initiative, i.e., putatively characterized metabolites against a single parameter such as molecular weight (MW) (Chaleckis et al., 2019), we annotated the differentially expressed metabolites according to the accurate MW by searching the exact MW against online Human Metabolome Database (HMDB) version 4.0 (Wishart et al., 2018) and Kyoto Encyclopedia of Genes and Genomes (KEGG) database (Wu et al., 2018). The differentially expressed metabolites identified from the above approach were used to perform pathway enrichment analysis using MetaboAnalyst 4.0. This allowed exploring upregulated and downregulated metabolic pathways in which the differential metabolites are involved in order to obtain an accurate insight into the underlying biology of the differentially expressed metabolites (Chong et al., 2018).

### Statistical Analysis

The UNIVARIATE procedure of SAS 9.4 (SAS Institute Inc., Cary, NC, United States) was used for body measurements between CON and MET groups at birth, cumulative starter NDF intake and cumulative BW gain. The MIXED procedure of SAS 9.4 was used for repeated measures analysis of body measurements, daily starter intake and average daily gain (ADG) at 14, 28, and 42 days of age. To avoid a potential confounding effect of initial BW on growth performance measures during the preweaning period, initial BW was included as a covariate in the mixed model ANOVA. All data are reported as covariate-adjusted means. Both maternal groups and time (day or week) were considered as fixed factors in the model, and the random effect was heifer calf. Comparison of bacterial alpha diversity indices in fecal microbial communities between CON and MET groups at birth was performed with the non-parametric Mann–Whitney unpaired *t*-test using JMP 13.2 (SAS Institute Inc., Cary, NC, United States). Permutational multivariate analysis of variance (PERMANOVA) utilizing a Bray–Curtis dissimilarity index (Anderson, 2001) was also run in JMP 13.2 (SAS Institute Inc., Cary, NC, United States) to determine differences in bacterial beta diversity indices between the two groups at 14, 28, and 42 days of age. Linear discriminant analysis (LDA) effect size (LEfSe) analysis was used to identify differentially abundant genera between CON and MET groups. Significance was determined at *P* ≤ 0.05 whereas tendencies were declared at *P* ≤ 0.10.

## Results

### Body Measurements and Growth Performance

At birth, neonatal heifer calves from cows fed MET had greater (*P* ≤ 0.05) BW, HH and WH (Table 2). Calves in the MET group tended to increase BW gain (*P* = 0.06) and cumulative BW gain (*P* = 0.08) (Table 3) despite a lack of difference in starter DMI (*P* = 0.77), cumulative starter NDF intake (*P* = 0.71), and overall FCR (*P* = 0.52). Along the same line, enhanced post-ruminal supply of methionine during late-pregnancy led to overall greater (*P* ≤ 0.05) BW and HH, and tended to increase WH (*P* = 0.06) during the preweaning period (Table 3). There was a clear effect of time (*P* < 0.01) on daily starter intake, ADG (Table 3) and body measurements (Table 3) during the preweaning period.

**TABLE 2:** Body measurements at birth in heifer calves born to cows offered a control diet (CON, *n* = 13) or CON supplemented with ethyl-cellulose rumen-protected methionine (MET, *n* = 13; Mepron^®^ at 0.09% of diet DM; Evonik Nutrition & Care GmbH, Germany) during last 28 days of pregnancy.

**Body measurement**	**CON**	**MET**	**SEM^1^**	***P*-value**
Body weight (kg)	40.08^b^	43.79^a^	0.91	0.04
Body length (cm)	109.30	109.90	1.57	0.80
Hip height (cm)	78.43^b^	82.32^a^	0.64	< 0.01
Hip width (cm)	16.14	15.66	0.39	0.43
Wither height (cm)	75.63^b^	78.17^a^	0.70	0.03

*^1^Standard error of the mean. ^*a,b*^Different letters indicate significant differences due to the main maternal effect (*P* < 0.05).*

**TABLE 3:** Body measurements and growth performance during preweaning period in heifer calves born to cows offered a control diet (CON, *n* = 13) or CON supplemented with ethyl-cellulose rumen-protected methionine (MET, *n* = 13; Mepron^®^ at 0.09% of diet DM; Evonik Nutrition & Care GmbH, Germany) during last 28 days of pregnancy.

	**Maternal**			***P*-value**		
**Week**	**CON**	**MET**	**SEM^1^**	**Maternal**	**Time**	**Maternal × Time**
**Weekly body weight (kg)**						
1	42.03	44.26	1.08	0.05	<0.01	0.15
2	44.56	46.76	1.08			
3	49.32	50.70	1.08			
4	54.87	56.24	1.08			
5	58.78	62.65	1.08			
6	63.33	67.57	1.08			
**Weekly body length (cm)**						
1	111.40	112.06	1.61	0.34	<0.01	0.29
2	114.09	116.32	1.61			
3	117.44	118.97	1.61			
4	121.07	122.25	1.61			
5	123.47	126.30	1.61			
6	126.73	130.15	1.61			
**Weekly hip height (cm)**						
1	80.31	83.19	0.71	0.02	<0.01	0.36
2	81.74	84.74	0.71			
3	83.32	85.65	0.71			
4	84.70	86.52	0.71			
5	86.61	88.25	0.71			
6	88.23	90.37	0.71			
**Weekly hip width (cm)**						
1	16.46	16.40	0.32	0.63	<0.01	0.81
2	17.84	17.70	0.32			
3	18.72	18.36	0.32			
4	19.33	19.05	0.32			
5	20.11	19.80	0.32			
6	20.77	20.84	0.32			
**Weekly wither height (cm)**						
1	76.90	78.59	0.69	0.06	<0.01	0.28
2	77.66	80.03	0.69			
3	79.75	81.47	0.69			
4	81.00	82.34	0.69			
5	82.49	84.16	0.69			
6	84.00	85.94	0.69			
**Weekly body weight gain (kg/d)^2^**						
1	0.02	0.25	0.10	0.10	<0.01	0.27
2	0.38	0.37	0.10			
3	0.69	0.55	0.10			
4	0.81	0.79	0.10			
5	0.57	0.91	0.10			
6	0.66	0.77	0.10			
Cumulative body weight gain (kg)	37.99	42.88	1.92	0.08		
**Weekly starter DMI (kg)**						
1	0.01	0.00	0.11	0.77	<0.01	0.97
2	0.07	0.02	0.11			
3	0.27	0.19	0.11			
4	0.40	0.42	0.11			
5	0.50	0.58	0.11			
6	0.99	1.06	0.11			
Cumulative starter DMI (kg)	15.01	15.87	2.16	0.77		
Cumulative starter NDF intake (kg)	5.97	6.21	0.45	0.71		
Overall FCR^3^	0.69	0.61	0.09	0.52		

*All data are reported as covariate-adjusted means. ^1^Pooled standard error of the mean. ^2^Average daily gain per week (kg) = (final BW – initial BW)/7. ^3^Overall FCR = cumulative starter DMI/cumulative body weight gain.*

### Fecal Microbiota at Birth

**FIGURE 1:**
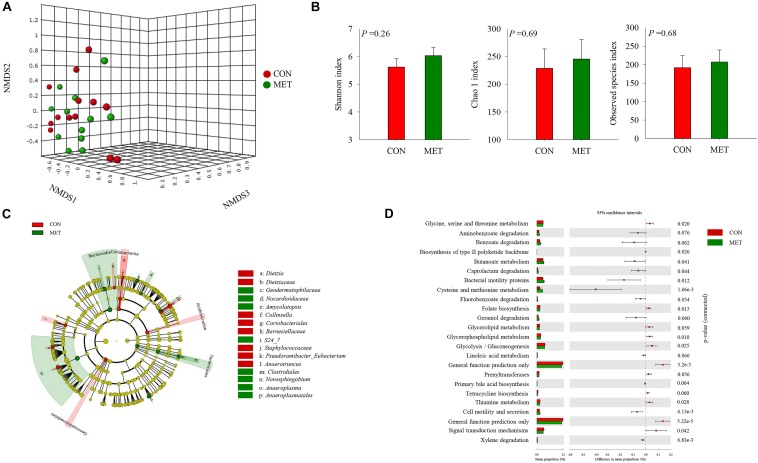
Fecal microbiota at birth in heifer calves born to cows offered a control diet (CON, *n* = 13) or CON supplemented with ethyl-cellulose rumen-protected methionine (MET, *n* = 13; Mepron^®^ at 0.09% of diet DM; Evonik Nutrition & Care GmbH, Germany) during the last 28 days of pregnancy. **(A)** Non-metric multidimensional scaling (NMDS) plot of fecal microbiota profile. **(B)** Alpha diversity indices. **(C)** Cladogram of LEfSe analysis shows the overrepresented microbial populations. Taxa are significant from LeFSe (*P* ≤ 0.05 and LDA cutoff > 3.0). **(D)** Histogram of the LDA scores reveals the most differentially regulated metabolic pathways in fecal microbiota at KEGG levels 3.

A total of 5,608,590 reads were retrieved from 104 sequenced samples and clustered into 6,494 ASVs (Supplementary Table S3). Despite a lack of difference in beta diversity of microbial communities (*P* = 0.45) between groups at birth detected with the NMDS approach (Figure 1A and Supplementary Figure S1), which were further confirmed by Shannon (*P* = 0.26), Chao 1 (*P* = 0.69) and observed species (*P* = 0.68) diversity indices (Figure 1B and Supplementary Figure S2), the LeFSe analysis revealed shifts in the fecal microbiota communities at birth in response to maternal methionine supply (Figure 1C and Table 4). For example, MET heifers had greater abundance (*P* ≤ 0.05 and LDA cutoff > 2.0) of *Geodermatophilaceae*, *Nocardioidaceae*, *Amycolatopsis*, *S24_7*, *Clostridiales*, *Novosphingobium*, *Anaeroplasma*, *Anaeroplasmatales*, but lower (*P* ≤ 0.05 and LDA cutoff > 2.0) *Dietzia*, *Dietziaceae*, *Collinsella*, *Coriobacteriales*, *Barnesiellaceae*, *Staphylococcaceae*, *Pseudoramibacter_Eubacterium* and *Anaerotruncus* (Figure 1C and Table 4). In addition, the MET microbiota was enriched with a greater number of functional genes (*P* ≤ 0.05) involved in methionine, cysteine and butanoate metabolism, caprolactam and xylene degradation, bacterial motility proteins, biosynthesis of type II polyketide backbone and cell motility and secretion (Figure 1D). In contrast, the microbiota of MET heifers had lower functional genes (*P* ≤ 0.05) for glycine, serine, threonine, thiamine and glycerophospholipid metabolism, folate biosynthesis, glycolysis/gluconeogenesis, signal transduction mechanisms and general function prediction.

**TABLE 4:** Relative abundance (%) of the overrepresented bacteria highlighted by LeFSe analysis (*P* ≤ 0.05 and LDA cutoff > 2.0) in feces at birth in heifer calves born to cows offered a control diet (CON, *n* = 13) supplemented with ethyl-cellulose rumen-protected methionine (MET, *n* = 13; Mepron^®^ at 0.09% of diet DM; Evonik Nutrition & Care GmbH, Germany) compared with heifer calves born to cows offered a control diet (CON, *n* = 13) during the last 28 days of pregnancy.

**Bacteria**	**CON**	**MET**
*Dietzia*	1.244^a^	0.177^b^
*Dietziaceae*	1.256^a^	0.197^b^
*Geodermatophilaceae*	0.000^b^	0.018^a^
*Nocardioidaceae*	0.047^b^	0.891^a^
*Amycolatopsis*	0.000^b^	0.217^a^
*Collinsella*	0.022^a^	0.001^b^
*Coriobacteriales*	0.041^a^	0.006^b^
*Barnesiellaceae*	0.015^a^	0.000^b^
*S24-7*	2.287^b^	3.837^a^
*Staphylococcaceae*	0.139^a^	0.004^b^
*Pseudoramibacter_Eubacterium*	0.066^a^	0.000^b^
*Anaerotruncus*	0.127^a^	0.005^b^
*Clostridiales*	2.087^b^	3.593^a^
*Novosphingobium*	0.000^b^	0.207^a^
*Anaeroplasma*	0.000^b^	0.130^a^
*Anaeroplasmatales*	0.000^b^	0.145^a^

*^*a,b*^Different letters indicate significant differences due to the main maternal effect (*P* < 0.05).*

### Fecal Microbiota During the Preweaning Period

**FIGURE 2:**
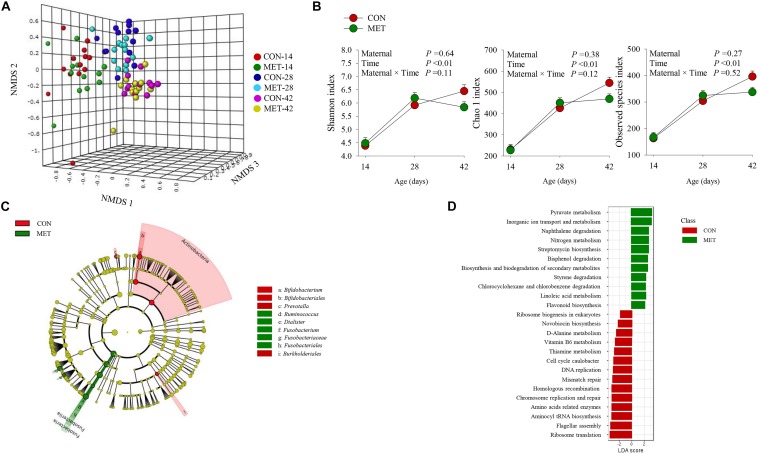
Fecal microbiota during preweaning period in heifer calves born to cows offered a control diet (CON, *n* = 13) or CON supplemented with ethyl-cellulose rumen-protected methionine (MET, *n* = 13; Mepron^®^ at 0.09% of diet DM; Evonik Nutrition & Care GmbH, Germany) during the last 28 days of pregnancy. **(A)** Non-metric multidimensional scaling (NMDS) plot of fecal microbiota profile. **(B)** Alpha diversity indices. **(C)** Cladogram of LEfSe analysis shows the overrepresented microbial populations. Taxa are significant from LeFSe (*P* ≤ 0.05 and LDA cutoff > 3.0). **(D)** Microbial functional predictions at KEGG levels 3.

The NMDS plot revealed no differences at the beta diversity level (*P* = 0.67) between MET and CON heifers at day 14, 28, and 42 of age (Figure 2A and Supplementary Figure S3). These results were further confirmed by Shannon (*P* = 0.64), Chao 1 (*P* = 0.38) and observed species (*P* = 0.27) diversity indices revealing no differences in fecal bacterial alpha diversity at any tested time-point (Figure 2B and Supplementary Figure S2). In addition, alpha diversity indices did not reveal maternal diet and time interactions over time (*P* > 0.10) (Figure 2B). LeFSe analysis for bacterial taxa revealed shifts in the preweaning microbiota communities in response to prenatal MET (Figure 2C and Table 5). For example, MET heifers had greater abundance (*P* ≤ 0.05 and LDA cutoff > 2.0) of *Ruminococcus*, *Dialister*, *Fusobacterium*, *Fusobacteriaceae*, *Fusobacteriales* but lower (*P* ≤ 0.05 and LDA cutoff > 2.0) *Actinomyces*, *Bifidobacterium*, *Bifidobacteriales*, *Prevotella*, *Paraprevotellaceae*, *Clostridium*, and *Burkholderiales* (Figure 2C and Table 5). In addition, the MET microbiota was enriched with a greater number of functional genes (*P* ≤ 0.05 and LDA cutoff > 2.0) involved in pyruvate, nitrogen, inorganic ion transport, naphthalene, bisphenol, styrene chlorocyclohexane and chlorobenzene degradation, streptomycin and flavonoid biosynthesis and biosynthesis and biodegradation of secondary metabolites (Figure 2D). In contrast, the microbiota of MET heifers had lower functional genes (*P* ≤ 0.05 and LDA cutoff > 2.0) for ribosome biogenesis in eukaryotes, novobiocin biosynthesis, D-alanine, thiamine and vitamin B6 metabolism, cell cycle caulobacter, DNA replication, mismatch repair, homologous recombination, chromosome replication and repair, amino acids related enzymes, aminocyl tRNA biosynthesis, flagellar assembly and ribosome translation.

**TABLE 5:** Relative abundance (%) of the overrepresented bacteria highlighted by LeFSe analysis (*P* ≤ 0.05 and LDA cutoff > 2.0) in feces during preweaning period in heifer calves born to cows offered a control diet (CON, *n* = 13) supplemented with ethyl-cellulose rumen-protected methionine (MET, *n* = 13; Mepron^®^ at 0.09% of diet DM; Evonik Nutrition & Care GmbH, Germany) compared with heifer calves born to cows offered a control diet (CON, *n* = 13) during the last 28 days of pregnancy.

**Bacteria**	**CON**	**MET**
*Bifidobacterium*	3.992^a^	2.749^b^
*Bifidobacteriales*	0.005^a^	0.000^b^
*Prevotella*	0.049^a^	0.030^b^
*Ruminococcus*	1.072^b^	3.031^a^
*Dialister*	0.000^b^	0.005^a^
*Fusobacterium*	19.822^b^	25.958^a^
*Fusobacteriaceae*	0.021^b^	0.063^a^
*Fusobacteriales*	0.058^b^	0.068^a^
*Burkholderiales*	0.044^a^	0.035^b^

*^*a,b*^Different letters indicate significant differences due to the main maternal effect (*P* < 0.05).*

### Fecal Metabolome at Birth

**FIGURE 3:**
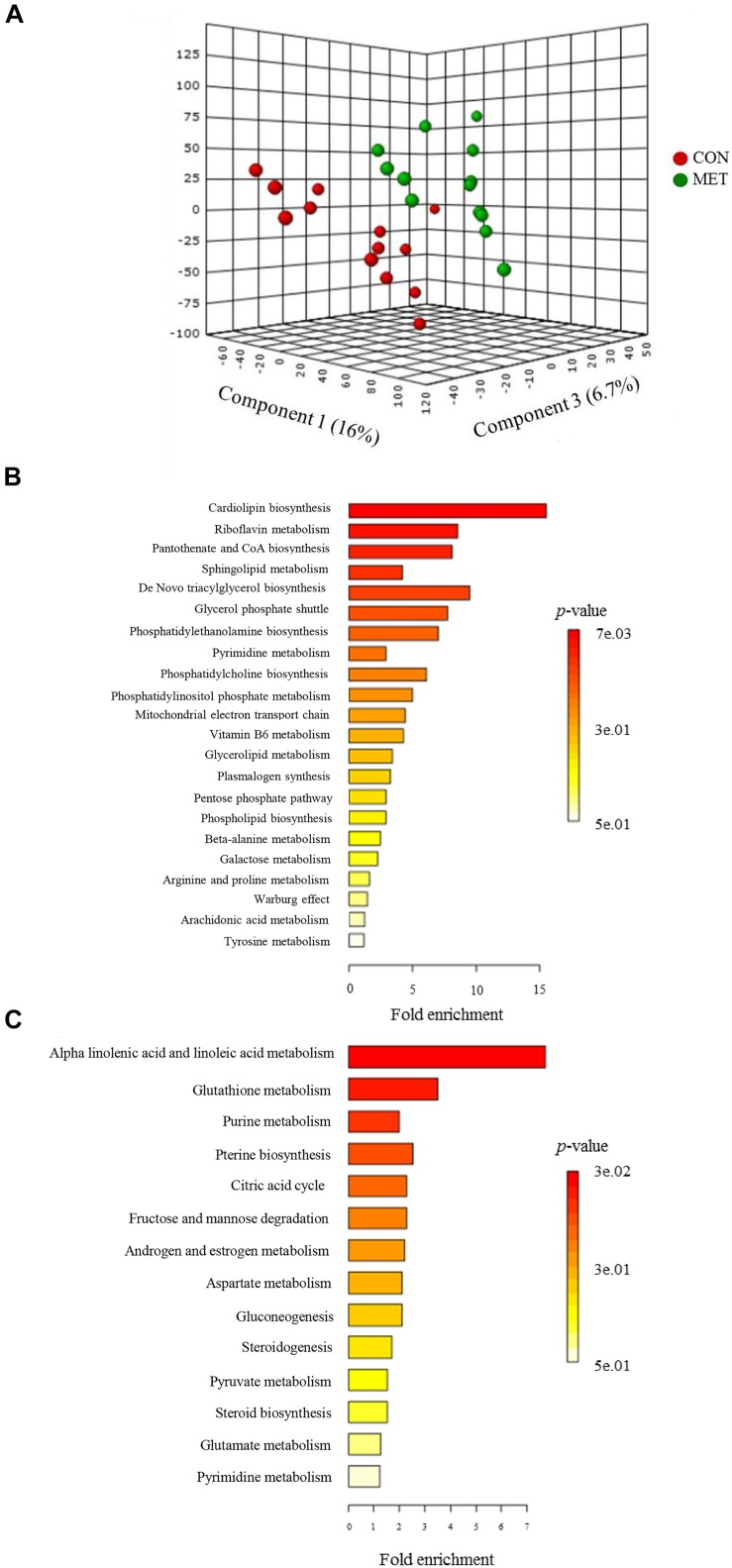
Fecal metabolome data at birth in heifer calves born to cows offered a control diet (CON, *n* = 13) or CON supplemented with ethyl-cellulose rumen-protected methionine (MET, *n* = 13; Mepron at 0.09% of diet DM; Evonik Nutrition & Care GmbH, Germany) during the last 28 days of pregnancy. **(A)** 3D scores plot of partial least square discriminant analysis (PLS-DA) model. **(B,C)** Upregulated and downregulated metabolic pathways in MET group at birth.

**FIGURE 4:**
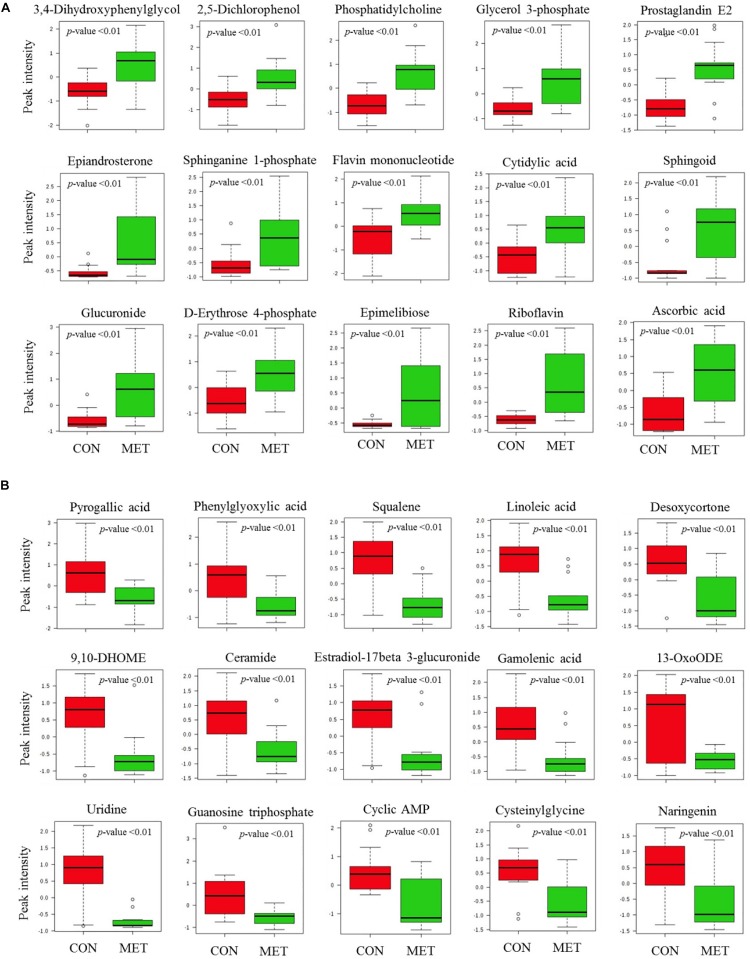
Fecal metabolites most strongly influencing discrimination by partial least square discriminant analysis (PLS-DA) model at birth in heifer calves born to cows offered a control diet supplemented with ethyl-cellulose rumen-protected methionine (MET; Mepron at 0.09% of diet DM; Evonik Nutrition & Care GmbH, Germany) during the last 28 days of pregnancy. **(A,B)** Upregulated and downregulated fecal metabolites in MET group at birth.

The PLS-DA plot of metabolomics data from fecal samples revealed a clear separation between MET and CON groups at birth (Figure 3A). A total of 30 differentially abundant metabolites were identified and annotated in fecal samples at birth (Figure 4 and Table 6). The MET heifers had a greater concentration (*P* < 0.01) of 3,4-dihydroxyphenylglycol, 2,5-dichlorophenol, phosphatidylcholine, glycerol 3-phosphate, prostaglandin E2, epiandrosterone, sphinganine 1-phosphate, flavin mononucleotide, cytidylic acid, sphingoid, glucuronide, D-erythrose 4-phosphate, epimelibiose, riboflavin, and ascorbic acid (Figure 4A and Table 6). The enrichment of these metabolites resulted in upregulation (*P* ≤ 0.05) of multiple biological pathways (Figure 3B) including cardiolipin biosynthesis, riboflavin metabolism, pantothenate and coenzyme A (CoA) biosynthesis, sphingolipid metabolism, *de novo* triacylglycerol biosynthesis, glycerol phosphate shuttle, phosphatidylethanolamine biosynthesis, pyrimidine metabolism, phosphatidylcholine biosynthesis, phosphatidylinositol phosphate metabolism, mitochondrial electron transport chain, vitamin B_6_ metabolism, plasmalogen synthesis, pentose phosphate pathway, phospholipid biosynthesis, beta-alanine metabolism, galactose metabolism, arginine and proline metabolism, warburg effect, arachidonic acid metabolism, and tyrosine metabolism. In contrast, MET heifers had lower concentrations (*P* < 0.01) of pyrogallic acid, phenylglyoxylic acid, squalene, linoleic acid, desoxycortone, 9,10-DHOME, ceramide, estradiol-17beta 3-glucuronide, gamolenic acid, 13-OxoODE, uridine, guanosine triphosphate, cyclic AMP, cysteinylglycine and naringenin (Figure 4B and Table 6). The decrease of these metabolites resulted in downregulation (*P* ≤ 0.05) of several biological pathways (Figure 3C) including alpha linolenic acid and linoleic acid metabolism, glutathione metabolism, purine metabolism, pterine biosynthesis, citric acid cycle, fructose and mannose degradation androgen and estrogen metabolism, aspartate metabolism, gluconeogenesis, steroidogenesis, pyruvate metabolism, steroid biosynthesis, glutamate metabolism and pyrimidine metabolism.

**TABLE 6:** Chemical taxonomy of metabolites most strongly influencing discrimination by the partial least squares discriminate analysis (PLS-DA) that were upregulated and downregulated in feces at birth in heifer calves born to cows offered a control diet (CON, *n* = 13) supplemented with ethyl-cellulose rumen-protected methionine (MET, *n* = 13; Mepron^®^ at 0.09% of diet DM; Evonik Nutrition & Care GmbH, Germany) compared with heifer calves born to cows offered a control diet (CON, *n* = 13) during the last 28 days of pregnancy, following the conditions of VIP > 1.0 and |*p*-(corr)| ≥ 0.5.

	**Name**	**Chemical taxonomy**	
		**Super class**	**Sub class**
Increased in MET	3,4-Dihydroxyphenylglycol	Benzenoids	Benzenediols
	2,5-Dichlorophenol	Benzenoids	Halobenzenes
	Phosphatidylcholine	Lipids and lipid-like molecules	Glycerophosphocholines
	Glycerol 3-phosphate	Lipids and lipid-like molecules	Glycerophosphates
	Prostaglandin E2	Lipids and lipid-like molecules	Eicosanoids
	Epiandrosterone	Lipids and lipid-like molecules	Androstane steroids
	Sphinganine 1-phosphate	Lipids and lipid-like molecules	Phosphosphingolipids
	Flavin mononucleotide	Nucleosides, nucleotides, and analogues	Flavin nucleotides
	Cytidylic acid	Nucleosides, nucleotides, and analogues	Pyrimidine ribonucleotides
	Sphingoid	Organic nitrogen compounds	Amines
	Glucuronide	Organic oxygen compounds	Carbohydrates and carbohydrate conjugates
	D-Erythrose 4-phosphate	Organic oxygen compounds	Carbohydrates and carbohydrate conjugates
	Epimelibiose	Organic oxygen compounds	Carbohydrates and carbohydrate conjugates
	Riboflavin	Organoheterocyclic compounds	Alloxazines and isoalloxazines
	Ascorbic acid	Organoheterocyclic compounds	Furanones
Decreased in MET	Pyrogallic acid	Benzenoids	Phenols and derivatives
	Phenylglyoxylic acid	Benzenoids	Benzoyl derivatives
	Squalene	Lipids and lipid-like molecules	Triterpenoids
	Linoleic acid	Lipids and lipid-like molecules	Lineolic acids and derivatives
	Desoxycortone	Lipids and lipid-like molecules	Hydroxysteroids
	9,10-DHOME	Lipids and lipid-like molecules	Fatty acids and conjugates
	Ceramide	Lipids and lipid-like molecules	Ceramides
	Estradiol-17beta 3-glucuronide	Lipids and lipid-like molecules	Steroidal glycosides
	Gamolenic acid	Lipids and lipid-like molecules	Lineolic acids and derivatives
	13-OxoODE	Lipids and lipid-like molecules	Lineolic acids and derivatives
	Uridine	Nucleosides, nucleotides, and analogues	Pyrimidine nucleosides
	Guanosine triphosphate	Nucleosides, nucleotides, and analogs	Purine ribonucleotides
	Cyclic AMP	Nucleosides, nucleotides, and analogues	Cyclic purine nucleotides
	Cysteinylglycine	Organic acids and derivatives	Amino acids, peptides, and analogues
	Naringenin	Phenylpropanoids and polyketides	Flavans

### Fecal Metabolome During the Preweaning Period

**FIGURE 5:**
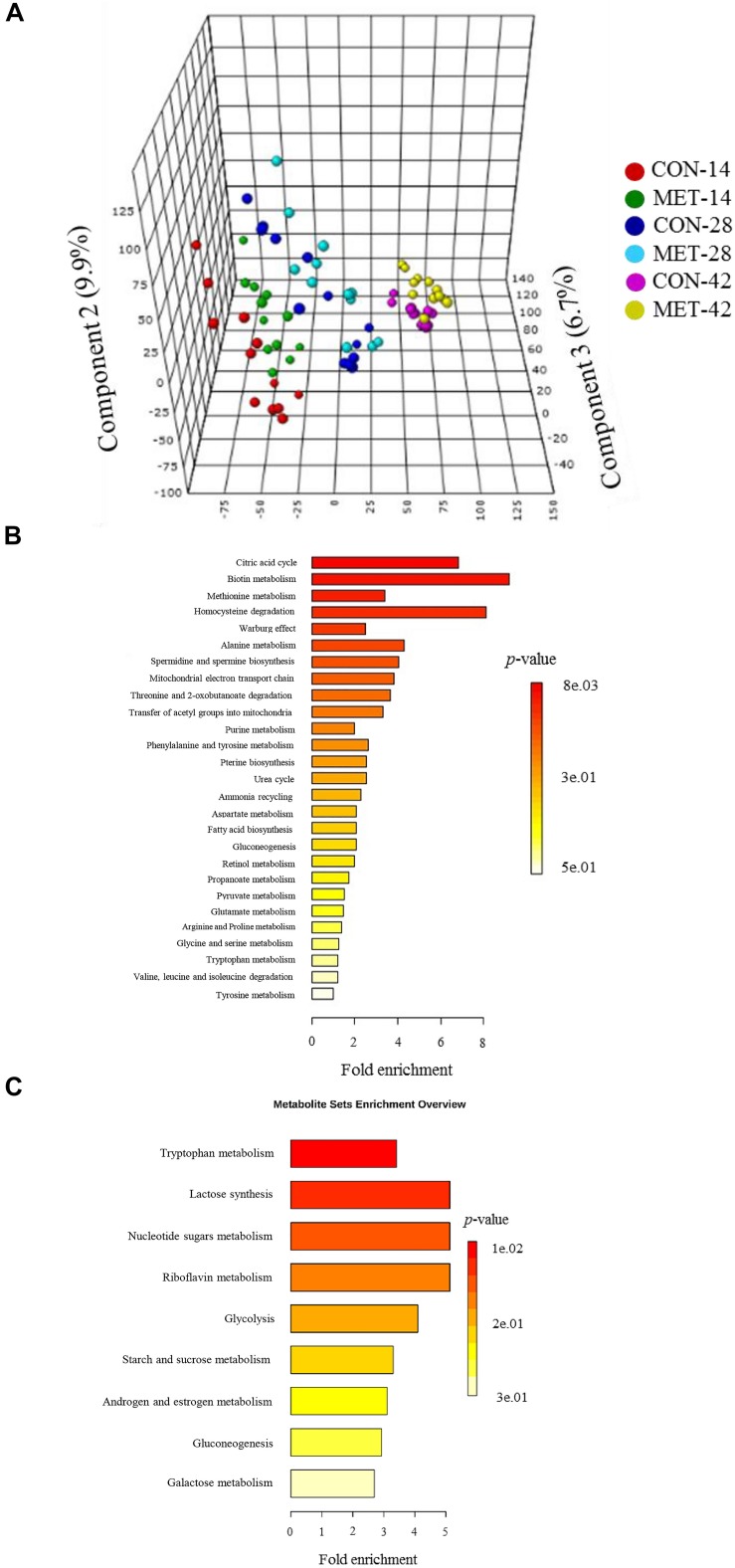
Fecal metabolome data during preweaning period in heifer calves born to cows offered a control diet (CON, *n* = 13) or CON supplemented with ethyl-cellulose rumen-protected methionine (MET, *n* = 13; Mepron at 0.09% of diet DM; Evonik Nutrition & Care GmbH, Germany) during the last 28 days of pregnancy. **(A)** 3D scores plot of partial least square discriminant analysis (PLS-DA) model. **(B,C)** Upregulated and downregulated metabolic pathways in MET group during preweaning period.

**FIGURE 6:**
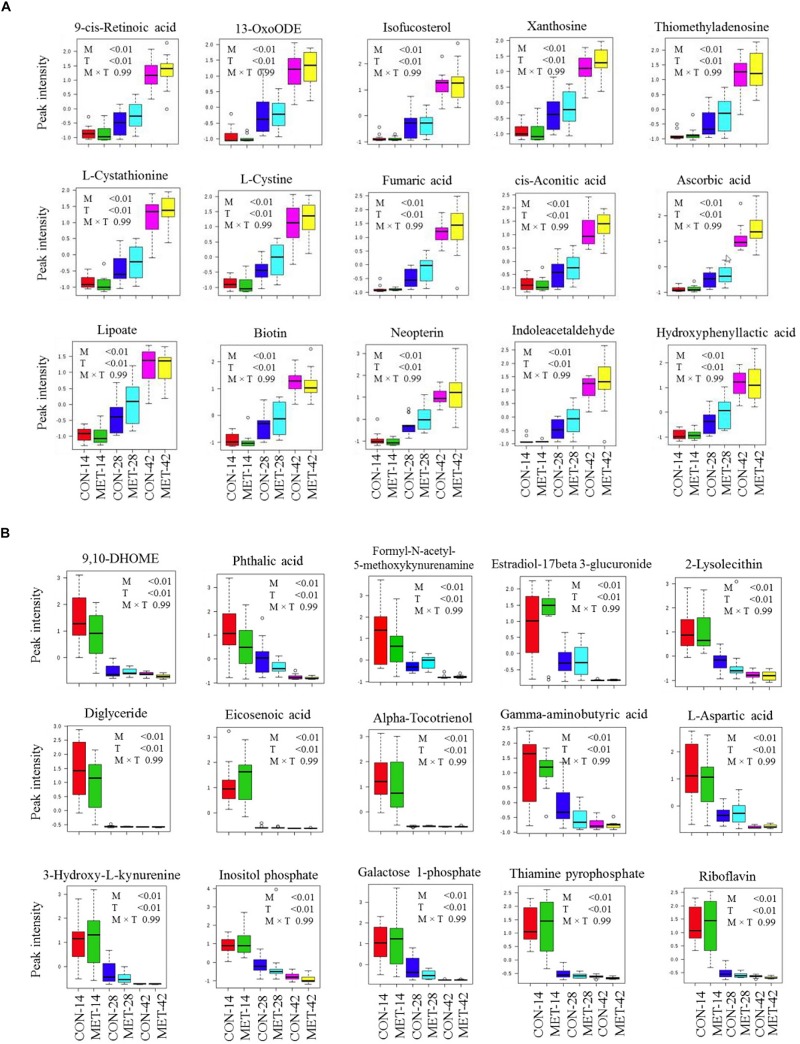
Fecal metabolites most strongly influencing discrimination by partial least square discriminant analysis (PLS-DA) model during preweaning period in heifer calves born to cows offered a control diet supplemented with ethyl-cellulose rumen-protected methionine (MET; Mepron at 0.09% of diet DM; Evonik Nutrition & Care GmbH, Germany) during the last 28 days of pregnancy. **(A,B)** Upregulated and downregulated fecal metabolites in MET group during preweaning period.

Differences in fecal metabolite profiles of MET and CON neonatal heifers at birth were revealed by the PLS-DA plot (Figure 5A). A total of 30 differentially abundant metabolites were identified and annotated in fecal samples during the preweaning period (Figure 6 and Table 7). Overall, MET heifers had a greater abundance (*P* < 0.01) of 9-*cis*-retinoic acid, 13-OxoODE, isofucosterol, xanthosine, thiomethyladenosine, L-cystathionine, L-cystine, fumaric acid, *cis*-aconitic acid, ascorbic acid, lipoate, biotin, neopterin, indoleacetaldehyde, and hydroxyphenyllactic acid (Figure 6A and Table 7). The enrichment of these metabolites resulted in the upregulation (*P* ≤ 0.05) of multiple biological pathways (Figure 5B) including citric acid cycle, biotin metabolism, methionine metabolism, homocysteine degradation, warburg effect, alanine metabolism, spermidine and spermine biosynthesis, mitochondrial electron transport chain, threonine and 2-oxobutanoate degradation, transfer of acetyl groups into mitochondria, purine metabolism, phenylalanine and tyrosine metabolism, pterine biosynthesis, urea cycle, ammonia recycling, aspartate metabolism, fatty acid biosynthesis, gluconeogenesis, retinol metabolism, propanoate metabolism, pyruvate metabolism, glutamate metabolism, arginine and proline metabolism, glycine and serine metabolism, tryptophan metabolism, valine, leucine and isoleucine degradation and tyrosine metabolism. In contrast, MET heifers had lower overall concentrations (*P* < 0.01) of 9,10-DHOME, phthalic acid, formyl-*N*-acetyl-5-methoxykynurenamine, estradiol-17beta 3-glucuronide, 2-lysolecithin, diglyceride, eicosenoic acid, alpha-tocotrienol, gamma-aminobutyric acid, L-aspartic acid, 3-hydroxy-L-kynurenine, inositol phosphate, galactose 1-phosphate, thiamine pyrophosphate and riboflavin (Figure 6B and Table 7). The decrease of these metabolites in Met calves resulted in downregulation (*P* ≤ 0.05) of several biological pathways (Figure 5C) including tryptophan metabolism, lactose synthesis, nucleotide sugars metabolism, riboflavin metabolism, glycolysis, starch and sucrose metabolism, androgen and estrogen metabolism, gluconeogenesis and galactose metabolism.

**TABLE 7:** Chemical taxonomy of metabolites most strongly influencing discrimination by the partial least squares discriminate analysis (PLS-DA) that were upregulated and downregulated in feces during preweaning period in heifer calves born to cows offered a control diet (CON, *n* = 13) supplemented with ethyl-cellulose rumen-protected methionine (MET, *n* = 13; Mepron^®^ at 0.09% of diet DM; Evonik Nutrition & Care GmbH, Germany) compared with heifer calves born to cows offered a control diet (CON, *n* = 13) during the last 28 days of pregnancy, following the conditions of VIP > 1.0 and |*p*-(corr)| ≥ 0.5.

	**Name**	**Chemical taxonomy**	
		**Super class**	**Sub class**
Increased in MET	9-*cis*-Retinoic acid	Lipids and lipid-like molecules	Retinoids
	13-OxoODE	Lipids and lipid-like molecules	Lineolic acids and derivatives
	Isofucosterol	Lipids and lipid-like molecules	Stigmastanes and derivatives
	Xanthosine	Nucleosides, nucleotides, and analogues	Purine nucleosides
	Thiomethyladenosine	Nucleosides, nucleotides, and analogs	5′-deoxy-5′-thionucleosides
	L-Cystathionine	Organic acids and derivatives	Amino acids, peptides, and analogs
	L-Cystine	Organic acids and derivatives	Amino acids, peptides, and analogs
	Fumaric acid	Organic acids and derivatives	Dicarboxylic acids and derivatives
	cis-Aconitic acid	Organic acids and derivatives	Tricarboxylic acids and derivatives
	Ascorbic acid	Organoheterocyclic compounds	Furanones
	(R)-Lipoate	Organoheterocyclic compounds	Lipoic acids and derivatives
	Biotin	Organoheterocyclic compounds	Biotin and derivatives
	Neopterin	Organoheterocyclic compounds	Pterins and derivatives
	Indoleacetaldehyde	Organoheterocyclic compounds	Indoles
	Hydroxyphenyllactic acid	Phenylpropanoids and polyketides	Phenylpropanoic acids
Decreased in MET	9,10-DHOME	Lipids and lipid-like molecules	Fatty acids and conjugates
	Phthalic acid	Benzenoids	Benzoic acids and derivatives
	Estradiol-17beta 3-glucuronide	Lipids and lipid-like molecules	Steroidal glycosides
	2-Lysolecithin	Lipids and lipid-like molecules	Glycerophosphocholines
	Diglyceride	Lipids and lipid-like molecules	Lineolic acids and derivatives
	Eicosenoic acid	Lipids and lipid-like molecules	Fatty acids and conjugates
	Alpha-Tocotrienol	Lipids and lipid-like molecules	Quinone and hydroquinone lipids
	Gamma-Aminobutyric acid (GABA)	Organic acids and derivatives	Amino acids, peptides, and analogs
	L-Aspartic acid	Organic acids and derivatives	Amino acids, peptides, and analogs
	3-Hydroxy-L-kynurenine	Organic oxygen compounds	Carbonyl compounds
	Inositol phosphate	Organic oxygen compounds	Alcohols and polyols
	Galactose 1-phosphate	Organic oxygen compounds	Carbohydrates and carbohydrate conjugates
	Formyl-*N*-acetyl-5-methoxykynurenamine	Organic oxygen compounds	Carbonyl compounds
	Riboflavin	Organoheterocyclic compounds	Alloxazines and isoalloxazines
	Thiamine pyrophosphate	Organoheterocyclic compounds	Pyrimidines and pyrimidine derivatives

## Discussion

### Body Measurements and Growth Performance

In a recent study using another cohort of cows from the present study (Batistel et al., 2017a), we reported that enhanced post-ruminal supply of methionine during late-pregnancy led to greater DMI, plasma methionine and plasma insulin. Furthermore, we discussed the possibility that those changes might have stimulated greater materno-fetal transfer of nutrients from maternal to fetal circulation via the upregulation of placental glucose-amino acid transporters and mammalian target of rapamycin (MTOR) signaling proteins. These placental adaptations to maternal methionine likely induced greater fetal growth during late-pregnancy (Sletmoen-Olson et al., 2000; Batistel et al., 2017a), which we confirmed when the entire cohort of calves was evaluated in terms of birth and preweaning BW, HH and WH in MET heifers (Alharthi et al., 2018). The greater BW, HH, WH along with greater BL and ADG during the preweaning period, i.e., through 42 days of age, in MET heifer calves in the present study agree with data reported on the entire cohort of calves (including male and female animals) (Alharthi et al., 2018). The lack of difference in cumulative starter DMI, cumulative NDF intake, and overall FCR between CON and MET heifer calves during the preweaning period also is in line with Alharthi et al. (2018).

The rumen in newborn calves is undeveloped at birth, thus, rapid development of the gastrointestinal tract during the preweaning period allows calves to transition into a mature ruminant (Heinrichs, 2005). When the rumen is underdeveloped, complex carbohydrates such as oligosaccharides and resistant starch are indigestible by enzymes in the small intestine of the calf and reach the hindgut where they are digested by microbial communities resulting in the production of energy substrates such as butyrate (Macfarlane and Englyst, 1986; Saulnier et al., 2009). Therefore, the hindgut microbiota is crucial for providing energy to the preweaned calf, influencing early development and health (Malmuthuge et al., 2014).

### Fecal Microbiota and Metabolome at Birth

Colonization of the bovine gut with microbes in early life is crucial to the development of mature metabolic functions, immune system and future health (Gomez et al., 2017). In addition, commensal bacteria in the hindgut protect the calf against pathogenic invasion (Kamada et al., 2013). The long-standing paradigm in embryology considers that the mammalian fetus develops in a sterile womb until birth (Welly et al., 2016). However, recent findings revealed that microbes are present in newborn calf meconium (i.e., the first feces at birth) (Alipour et al., 2018), suggesting that microbial colonization of the bovine hindgut might begin before birth and that prenatal dam-to-fetus efflux of commensal bacteria exists *in utero* via transmission through placental barriers (Funkhouser and Bordenstein, 2013). In support of this notion, *Enterococci* bacteria administered orally to pregnant mice were detected in fetal hindgut (Jiménez et al., 2008). Bacteria have also been detected in bovine placenta of healthy cows (Moore et al., 2017). In the current study, the detection of hindgut bacteria in newborn calves immediately at birth before colostrum feeding supports the idea that dam-to-fetus efflux of bacteria occurs in cattle.

The current study demonstrated that maternal methionine induced several shifts in hindgut microbiota and metabolome in newborn calves at birth. For example, the induction of several energy-related pathways in MET newborn fecal metabolome indicated a potential benefit to colonocytes. The induction of cardiolipin, riboflavin (vitamin B_2_), arginine and mitochondrial electron transport chain in MET neonates might have increased energy production via promoting mitochondrial membrane stability, respiratory chain and oxidative phosphorylation (Kajiwara et al., 2012; Adebayo et al., 2017). In addition, the upregulation of energy-related pathways such as pentose phosphate (PP), pantothenate (Vitamin B_5_), β-alanine, CoA, galactose, triacylglycerol and glycerol phosphate shuttle in MET neonates could have enhanced the capacity of the microbiota to readily-metabolize nutrients from colostrum reaching the hindgut (Wolfe, 2015). As such, the microbiota itself could have benefited or it could have provided intestinal cells and even organs of the calf (e.g., liver) with substrates for metabolism to support growth (Fernie et al., 2004; Coelho et al., 2015; Wang et al., 2017).

Reactive oxygen species (ROS) are key factors contributing to oxidative stress in cattle because they oxidize vital cellular components such as DNA, lipids and proteins, causing oxidative damage and harmful effects to the animal (Ling et al., 2018). As a result, inducing oxidative stress in calves during the preweaning period could impair growth performance and increase disease susceptibility (Gaál et al., 2006). Therefore, the development of strategies to enhance the antioxidant defense system and reduce oxidative stress in neonatal calves is an important goal. The upregulation of methionine metabolism and phosphatidylcholine biosynthesis in MET calves at birth agrees with data from Lin and Wang (2017). Those authors reported that supplemental dietary methionine induced the production of *S*-adenosyl methionine (SAM) and phosphatidylcholine biosynthesis by the gut microbiota of worms. Glutathione is an essential antioxidant, thus, downregulation of the glutathione pathway in the offspring in response to maternal methionine could reflect a lower state of oxidative stress in MET calves (Zhu C. et al., 2018). Because of its benefit against oxidative stress in non-ruminant cells, the induction of plasmalogen in MET calves might help reduce oxidative damage to the hindgut (Hossain et al., 2013). These data highlight the protective role of maternal methionine against oxidative damage in the hindgut of MET newborns. Although the exact mechanisms are unknown, biomarker profiling has confirmed the benefit of greater post-ruminal supply of methionine to dairy cows in terms of reducing oxidative stress in the circulation, liver and mammary gland (Vailati-Riboni et al., 2017; Batistel et al., 2018; Han et al., 2018).

The positive effect of maternal methionine on the enrichment of *Nocardioidaceae* and *Amycolatopsis* in the hindgut, well-known bacteria for producing antibiotics, underscores the protective effect of maternal methionine against pathogens in newborn hindgut. The family *Nocardioidaceae* produces siderophores to prevent growth of opportunistic fungi (Huang et al., 2014). The genus *Amycolatopsis* generates antibiotic compounds such as rifamorpholines and macrotermycins (Beemelmanns et al., 2017; Xiao et al., 2017), which would reduce pathogen colonization in the hindgut. In addition, the overrepresentation of polyketide biosynthesis in MET heifers supports our speculation that the production of antibiotics against selective pathogens was enhanced in response to the maternal exposure to methionine because polyketides are involved in the biosynthesis of several antibiotics such as streptomycin (Shestov et al., 2015). Greater production of antibiotics in the hindgut of MET newborns might reduce the presence of pathogenic bacteria such as *Collinsella* and *Anaerotruncus* (Chen et al., 2016; Zhang et al., 2017) observed in the MET group. The results above suggest that greater amounts of naturally-produced antibiotics at birth and during early life might enhance hindgut health resulting in better assimilation of nutrients that by-pass the small intestine. As such, because the rumen develops slowly after birth, hindgut function appears particularly important in these young animals. This notion is supported by data from the larger cohort of male and female calves from the present study in which MET compared with CON calves had lower fecal score, i.e., less diarrhea prevalence during the preweaning period (Alharthi et al., 2018). The greater BW, HH, WH along with greater BL and ADG during the preweaning period, i.e., through 42 days of age, in MET heifer calves in the present study agree with data reported on the entire cohort of calves (including male and female animals) (Alharthi et al., 2018). In this study, and because of the absence of differences in feed intake, these functional adaptations in the hindgut might help explain the better growth performance and nutrient utilization in MET calves.

The hindgut enrichment at birth in MET calves with microbial genes and metabolites regulating vital metabolic pathways that are typically associated with healthy than diseased states is surprising. For example, and similar to studies of inflammatory bowel disease (IBD) (Morgan et al., 2012; Fazlollahi et al., 2018), the induction of β-alanine and butanoate metabolism and bacterial motility and secretion in MET calves indicated a healthier status. Similarly, the upregulation of caprolactam and xylene degradation in the MET group revealed by PICRUSt functional analysis underscores the greater capacity of the microbiota to remove toxic caprolactam and xylene compounds from the hindgut (Hanson et al., 2016; Salimi et al., 2017). The suppression of glutamate and pterine pathways in MET newborns provides additional evidence of a healthier state in MET calves because previous studies reported that glutamate and pterine decreased in healthy humans compared with diseased counterparts with IBD, phenylketonuria and alcoholic liver cirrhosis (Pinheiro de Oliveira et al., 2016; Kolho et al., 2017).

Some published data in rodents offer support for microbial-derived sphingolipids and arachidonic acid in the gut as important factors to promote hindgut integrity, function and development during early life. For instance, sphingolipids are complex lipids known for their vital role as structural components of cell membranes and activators for natural killer T cells in the hindgut (An et al., 2014; Hasegawa et al., 2017). Because sphingolipids are crucial for the integrity of hindgut mucosa to prevent pathogenic microbe translocation into the calf circulation (Rusconi et al., 2018), the greater abundance of these lipids in MET calves could have enhanced their availability for transport into colonocytes. Arachidonic acid transport into hindgut cells in MET neonates also could have served an important role as an immune signaling molecule (Hwang, 1989). Together, these alterations would have rendered the hindgut in MET calves better able to cope with the consumption of colostrum and reduce pathogenic invasion, resulting in better hindgut functionality at birth.

Together, results suggest a potential prebiotic role for methionine supply during pregnancy on the hindgut including greater supply of energy-generating compounds, better antioxidant capacity, greater antibiotic production and overall healthier status at birth. Along with the greater birth body mass, the above results suggest that MET calves were in a more robust condition for facing the extrauterine environment. We speculate that the greater DMI in pregnant cows in response to the increase in post-ruminal supply of methionine played a role in determining the fecal microbiota and metabolome at birth (Batistel et al., 2017a). Because we performed the hindgut sampling using a deep fecal swab (10 cm) immediately after birth and before feeding colostrum, it is unlikely that external microorganisms would have affected the microbiota. Therefore, the detection of bacteria in the hindgut of newborns provides evidence for the existence of dam-to-fetus transmission *in utero*.

### Fecal Microbiota and Metabolome During the Preweaning Period

Maternal methionine induced greater energy production in MET heifers during the preweaning period. For example, MET heifers had greater butyrate-producing bacteria such as *Ruminococcus* and *Fusobacterium* suggesting a better capacity for generating this important VFA in the hindgut (Moen et al., 2016; Clark et al., 2018). It is well-known that butyrate is a key fuel source for colonocytes, with an overall positive effect on hindgut function (Clausen and Mortensen, 1995; Bashiardes et al., 2016). Although VFA in the hindgut were not measured in the present study, the greater numbers of microbial genes in pathways such as pyruvate and propanoate metabolism, TCA cycle, mitochondrial electron transport chain and fatty acid biosynthesis in MET calves support the notion that maternal methionine promoted the colonization of bacteria capable of fermenting indigestible matter reaching the hindgut.

Metabolome data demonstrated that maternal methionine induced the metabolism of essential vitamins such as biotin (vitamin B_7_). It is well-established that cattle cannot synthesize biotin, thus, must obtain it from microbes (rumen, hindgut) harboring the enzymatic capacity for its synthesis (Hayashi et al., 2017). This vitamin is an essential cofactor for various enzymes required for the metabolism of glucose, fatty acids and amino acids (Rodriguez-Melendez and Zempleni, 2003). In addition, it regulates several cellular functions such as histone modifications, cell signaling and mucosal immune response (Jenkins et al., 2017). These hindgut adaptations in response to maternal methionine supply would stimulate the generation of extra sources of energy to meet the calf’s needs. Because calves in the MET and CON group had similar starter intake during the entire preweaning period, alterations of hindgut microbiota and metabolome profiles associated with greater maternal supply of methionine are impressive. Taking into account the observed microbiota and metabolome profiles between Met and CON calves at birth it would appear that the beneficial profiles were established prior to solid feed intake. Subsequently, microbial populations in MET calves appeared to have had a greater capacity to maximize energy generation from the same amount of feed intake.

Maternal methionine led to enrichment of several amino acids such as methionine, arginine, proline, alanine, phenylalanine, tryptophan and tyrosine along with inducing the degradation of valine, leucine and isoleucine in the hindgut. Arginine and proline are important for normal cellular function and growth via their involvement in DNA and RNA synthesis, protein glycosylation and detoxification (Sparks, 2014; Li H.H. et al., 2018). Methionine and arginine are required for the synthesis of spermidine and spermine (Leruez et al., 2018), hence, the induction of spermidine and spermine biosynthesis observed in MET heifers in the current study was likely associated with the upregulation of methionine and arginine metabolism. In rodents, spermidine and spermine are essential for protecting cells against oxidative damage (Minois et al., 2011). In addition, tyrosine and two of its metabolites, *p*-hydroxyphenylacetic and cinnamic acids, can decrease the production of ROS (Beloborodova et al., 2012). Therefore, by preventing oxidative damage, the induction of spermidine, spermine and tyrosine in the MET group could have contributed to maintaining hindgut integrity. As such, the efficiency of hindgut utilization of microbial-derived compounds during the preweaning period would have been optimized.

Alanine is an important gluconeogenic amino acid in dairy cows (Aschenbach et al., 2010). The degradation of branched-chain amino acids (BCAA, valine, leucine, isoleucine) produces acetyl-CoA and succinyl-CoA that are important in energy-related pathways (Harper et al., 1984). In addition, BCAA catabolism generates branched-chain α-keto acids that activate MTOR, a major nutrient signaling pathway regulating cellular growth (Avruch et al., 2009). Therefore, the upregulation of alanine and the degradation of BCAA in MET heifers might have enhanced availability of metabolically-important compounds for the calf. Phenylalanine, tryptophan and tyrosine are precursors of neurotransmitters including dopamine and serotonin (Bergwerff et al., 2016), thus, the increase in these amino acids in dairy heifers in response to maternal methionine could have potentially enhanced gut-brain crosstalk in preweaned MET heifers. Although the potential for these metabolites for absorption from the hindgut cannot be discounted, it is also likely that microbes in the hindgut could have metabolize them further. For instance, members of the *Peptostreptococcus* genus consume glutamate and tryptophan (Lin et al., 2017), effectively limiting their availability to colonocytes. Further studies are needed to understand relationships between the hindgut microbiota and amino acid metabolism in ruminants.

Microbiota in MET calves during the preweaning period was enriched with a greater number of functional genes involved in antibiotic biosynthesis such as streptomycin and was in line with the proliferation of antibiotic-producing bacteria at birth (*Nocardioidaceae* and *Amycolatopsis*). The release of naturally-produced antibiotics in the hindgut during the preweaning period could have had a positive effect in the control of pathogenic microbe colonization, thereby contributing to hindgut health and function. This would have been especially important due to the high susceptibility of young calves to digestive disorders such as diarrhea (Smith, 2015). Such effect would have contributed to the positive impact of maternal methionine on preweaning growth performance. Additional evidence for beneficial effects of maternal methionine supply on the promotion of hindgut health in MET heifers could be discerned by the greater abundance of *Dialister* and lower *Actinomyces, Prevotella*, and *Burkholderiales*. The enrichment of genus *Dialister* in the human hindgut is a marker of good health status (Tito and Cypers, 2017). In humans, Del Chierico et al. (2018) reported lower abundance of genus *Actinomyces* in the hindgut of healthy compared with obese patients. In rodents and humans, the genus *Prevotella* is less abundant in healthy versus inflamed hindgut (Dillon et al., 2016; Su et al., 2018). Order *Burkholderiales* declined in hindgut of healthy compared with individuals experiencing inflammation (Stanisavljević et al., 2016). The lower bacterial genes responsible for flagellar assembly in the MET calf microbiota also supports the view of better hindgut health in response to maternal methionine. Flagellin is involved in bacterial flagellar filament structure and the rotation of these filaments provides bacteria with motility capacity (Silverman and Simon, 1974). Studies in humans and mice demonstrated that decreasing bacterial motility in the hindgut is a feature of a healthier gut compared with colitis or IBD (Lodes et al., 2004). Therefore, the above results are consistent with a better hindgut health status in response to enhanced maternal post-ruminal supply of methionine.

The present study utilized an integrative approach through a combination of high-throughput microbiomics and untargeted metabolomics to generate the first insights into changes in hindgut microbiota, its metabolites and their relationship with various physiologic aspects in neonatal dairy calves. These data provide evidence that enhanced maternal post-ruminal supply of methionine induced a shift in the microbiota and metabolome of the offspring toward a more efficient profile, both in terms of helping reduce populations of microbial pathogens and enhance production of key nutrients such as essential amino acids and vitamins. As such, the hindgut of calves exposed to greater methionine supply *in utero* appeared more efficient at deriving nutrients to sustain greater body mass and growth performance in the preweaning and early postweaning periods. Together, data revealed fundamental mechanisms about the role of post-ruminal maternal methionine supply during late-gestation for enhancing fetal development and postnatal growth performance. These findings indicate that methionine supplementation may well be an excellent candidate for dietary programming of the calf microbiota as maternal prebiotic. Additional research in this area to clarify the underlying mechanisms appears warranted.

## Author Contributions

JL, CP, and AH conceived and designed the experiments. AE and AA managed the calves and collected the performance data. AE harvested the biological samples, performed the lab analyses, metabolome analysis, and statistical analysis of calf data, and wrote the manuscript. AE and MZ performed the microbiota analysis. All authors read and approved the final version of the manuscript.

## Conflict of Interest Statement

CP and AH are employees of Evonik Industries (Evonik Nutrition & Care GmbH, Hanau-Wolfgang, Germany), which had a role in the study design and provided financial support to cover costs of animal use, data collection, and sample analyses. The remaining authors declare that the research was conducted in the absence of any commercial or financial relationships that could be construed as a potential conflict of interest.

## Supplementary Material

FIGURE S1 Phyla level taxonomic distribution in fecal samples at birth in heifer calves born to cows offered a control diet (CON, *n* = 13) or CON supplemented with ethyl-cellulose rumen-protected methionine (MET, *n* = 13; Mepron^®^ at 0.09% of diet DM; Evonik Nutrition & Care GmbH, Germany) during the last 28 days of pregnancy.

FIGURE S2 Rarefaction analysis of the fecal microbiota at birth and during preweaning period in heifer calves born to cows offered a control diet (CON, *n* = 13) supplemented with ethyl-cellulose rumen-protected methionine (MET, *n* = 13; Mepron^®^ at 0.09% of diet DM; Evonik Nutrition & Care GmbH, Germany) compared with heifer calves born to cows offered a control diet (CON, *n* = 13) during the last 28 days of pregnancy.

FIGURE S3 Phyla level taxonomic distribution in fecal samples during preweaning period at day 14, 28, and 42 of age in heifer calves born to cows offered a control diet (CON, *n* = 13) or CON supplemented with ethyl-cellulose rumen-protected methionine (MET, *n* = 13; Mepron^®^ at 0.09% of diet DM; Evonik Nutrition & Care GmbH, Germany) during the last 28 days of pregnancy.

TABLE S1 Ingredient and nutrient composition of far-off (−45 to −29 days relative to parturition) and close-up (from −28 days to parturition) maternal diets.

TABLE S2 Nutrient composition (mean ± standard deviation) and diet evaluation using NRC (2001) of maternal diets fed to multiparous Holstein cows prepartum.

TABLE S3 Number of 16S rRNA amplicon sequences (±standard deviation) in feces at birth and during preweaning period in heifer calves born to cows offered a control diet (CON, *n* = 13) supplemented with ethyl-cellulose rumen-protected methionine (MET, *n* = 13; Mepron^®^ at 0.09% of diet DM; Evonik Nutrition & Care GmbH, Germany) compared with heifer calves born to cows offered a control diet (CON, *n* = 13) during the last 28 days of pregnancy.
