# Supplementary figures and images for: Supply of Methionine During Late-Pregnancy Alters Fecal Microbiota and Metabolome in Neonatal Dairy Calves Without Changes in Daily Feed Intake
Source: Front Microbiol. 2019 Sep 19;10:2159. doi: 10.3389/fmicb.2019.02159 (PMC6761860; doi:10.3389/fmicb.2019.02159)

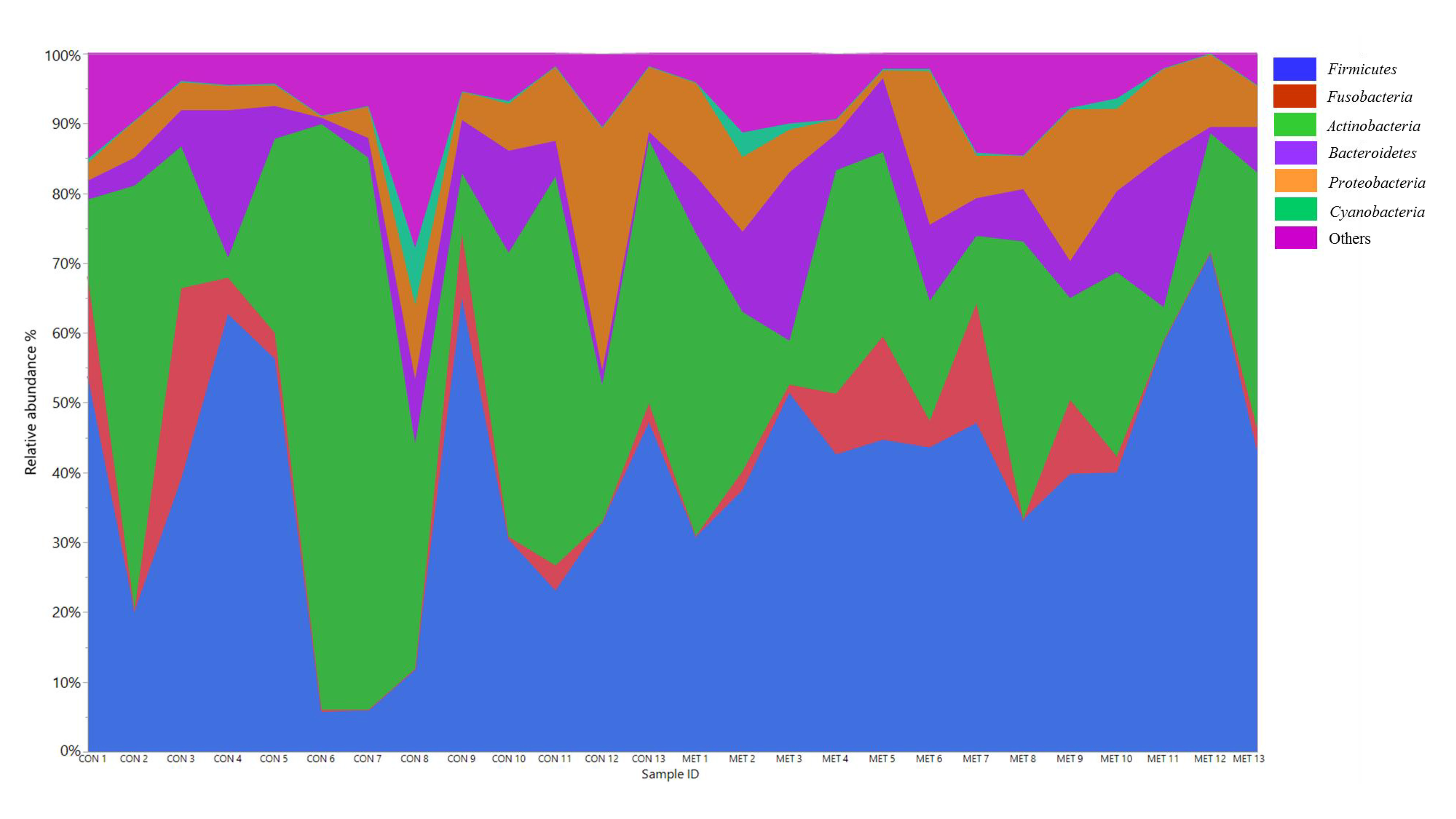

Supplement: FIGURE S1 — Phyla level taxonomic distribution in fecal samples at birth in heifer calves born to cows offered a control diet (CON, n = 13) or CON supplemented with ethyl-cellulose rumen-protected methionine (MET, n = 13; Mepron® at 0.09% of diet DM; Evonik Nutrition & Care GmbH, Germany) during the last 28 days of pregnancy. [file Image_1.JPEG]

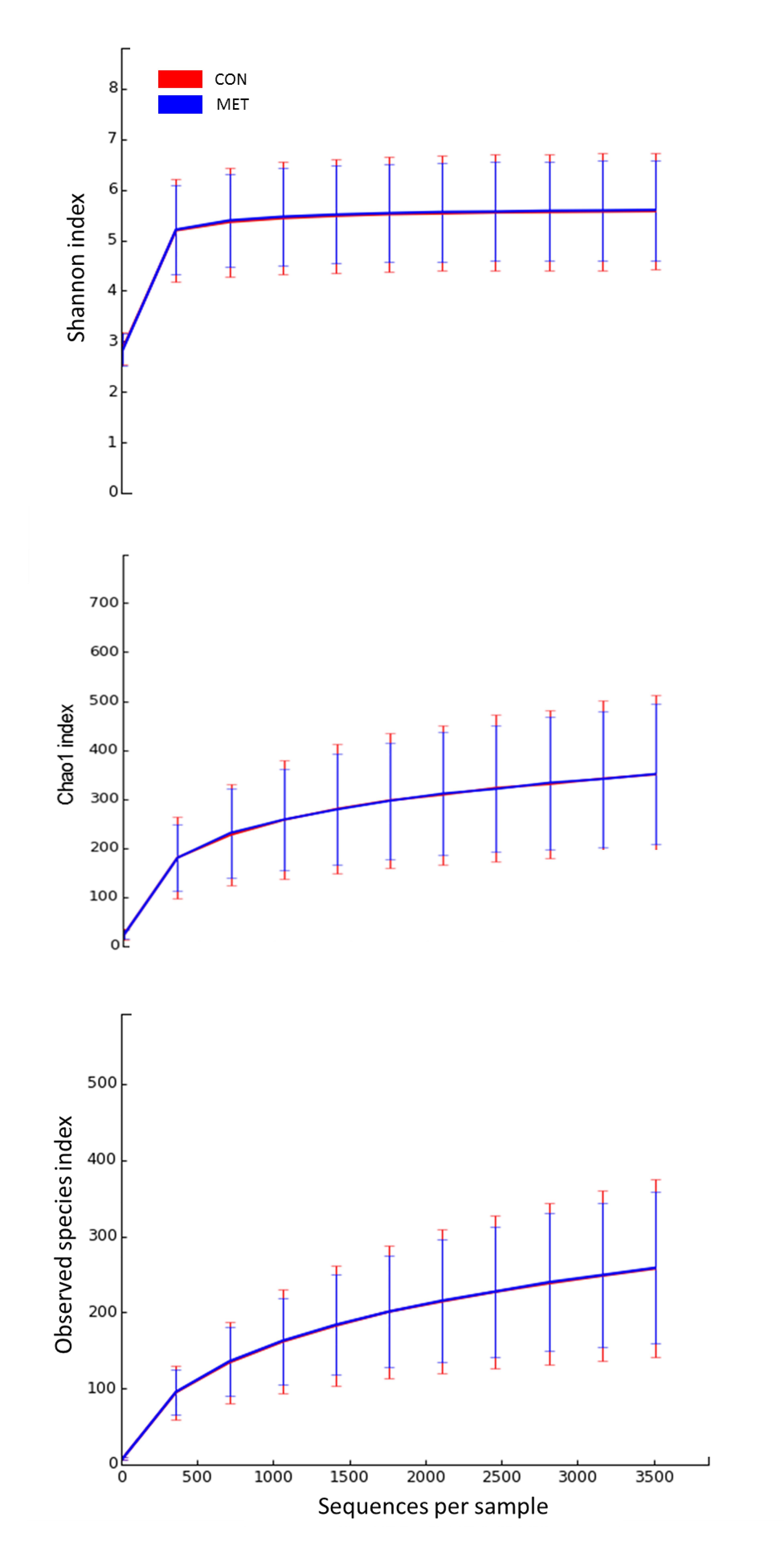

Supplement: FIGURE S2 — Rarefaction analysis of the fecal microbiota at birth and during preweaning period in heifer calves born to cows offered a control diet (CON, n = 13) supplemented with ethyl-cellulose rumen-protected methionine (MET, n = 13; Mepron® at 0.09% of diet DM; Evonik Nutrition & Care GmbH, Germany) compared with heifer calves born to cows offered a control diet (CON, n = 13) during the last 28 days of pregnancy. [file Image_2.JPEG]

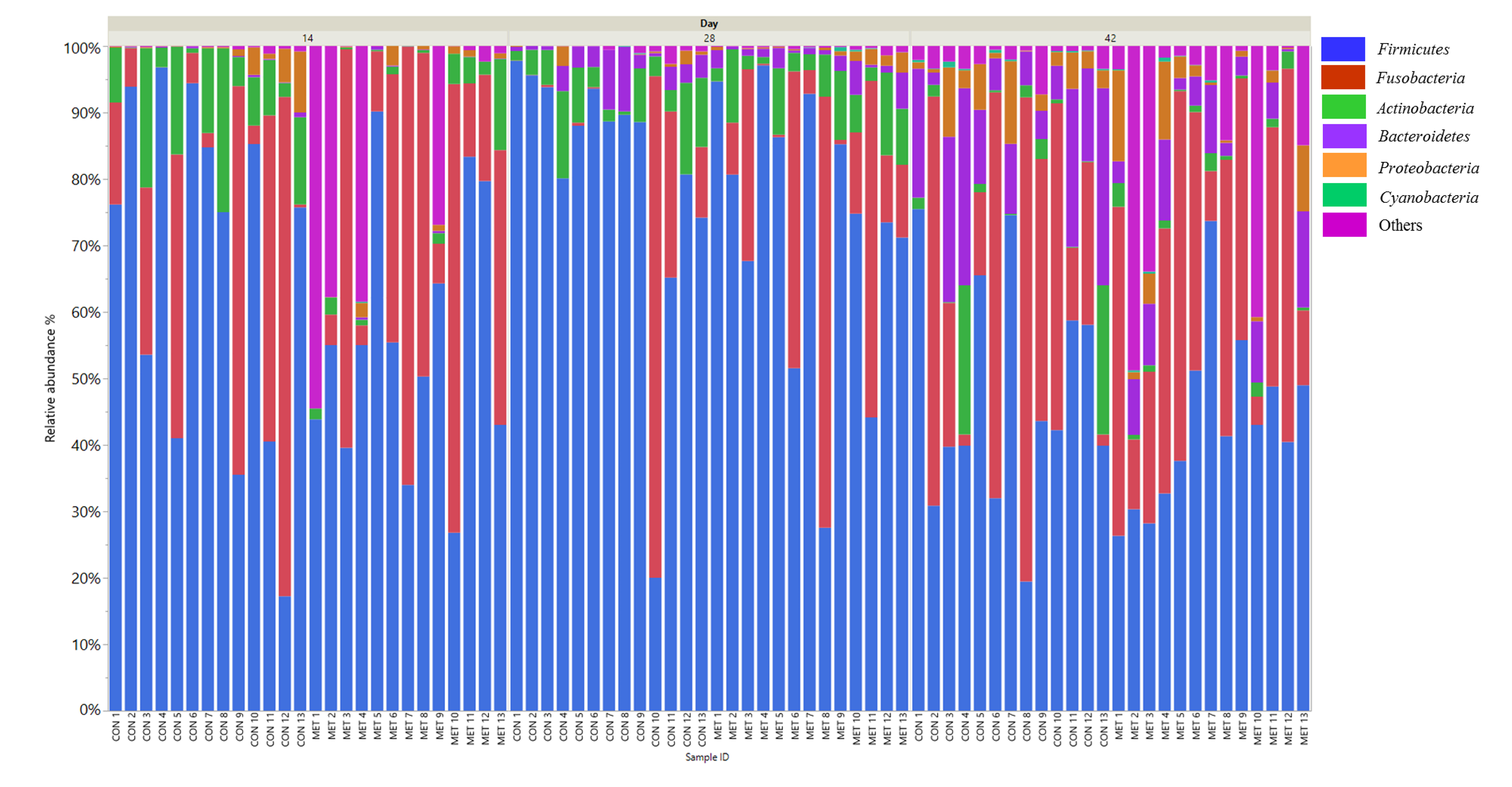

Supplement: FIGURE S3 — Phyla level taxonomic distribution in fecal samples during preweaning period at day 14, 28, and 42 of age in heifer calves born to cows offered a control diet (CON, n = 13) or CON supplemented with ethyl-cellulose rumen-protected methionine (MET, n = 13; Mepron® at 0.09% of diet DM; Evonik Nutrition & Care GmbH, Germany) during the last 28 days of pregnancy. [file Image_3.JPEG]
