# Supplementary material for: Supply of Methionine During Late-Pregnancy Alters Fecal Microbiota and Metabolome in Neonatal Dairy Calves Without Changes in Daily Feed Intake
Source: Front Microbiol. 2019 Sep 19;10:2159. doi: 10.3389/fmicb.2019.02159 (PMC6761860; doi:10.3389/fmicb.2019.02159)
Supplement: TABLE S1 — Ingredient and nutrient composition of far-off (−45 to −29 days relative to parturition) and close-up (from −28 days to parturition) maternal diets. [file Table_1.docx]

*Supplementary Material*

**Supply of Methionine During Late-Pregnancy Alters Fecal Microbiome and Metabolome in Neonatal Dairy Calves without Changes in Daily Feed Intake**

Ahmed Elolimy^1,2^, Mohamed Zeineldin^3,4^, Abdulrahman Alharthi^1,2^, Claudia Parys^5^, Ariane Helmbrecht^5^, Juan J. Loor^1,2,6,*^

^1^Mammalian NutriPhysioGenomics, Department of Animal Sciences, University of Illinois, Urbana, Illinois, USA. ^2^Department of Animal Sciences, University of Illinois, Urbana, IL. ^3^Integrated Food Animal Management Systems, Department of Veterinary Clinical Medicine, University of Illinois, Urbana, Illinois, USA. ^4^Department of Animal Medicine, College of Veterinary Medicine, Benha University, Egypt. ^5^Evonik Nutrition & Care GmbH, Hanau-Wolfgang, Germany. ^6^Division of Nutritional Sciences, Illinois Informatics Institute, University of Illinois, Urbana, Illinois, USA.

***Correspondence:** Dr. Juan J. Loor, jloor@illinois.edu

**TABLE S1.** Ingredient and nutrient composition of far-off (−45 d to −29 d relative to parturition) and close-up (from −28 d to parturition) maternal diets.

| **Ingredient, % of DM** | **Far-off** | **Close-up** |
| --- | --- | --- |
| Alfalfa haylage | — | 6.55 |
| Corn silage | 34.7 | 26.6 |
| Wheat straw | 33.7 | 26.5 |
| Corn grain, ground, dry | — | 12.6 |
| Cottonseed | — | — |
| Molasses, beet sugar | — | 4.03 |
| Soybean hulls | 15.7 | 3.46 |
| Soybean meal, 48% CP | 12.0 | 7.83 |
| Expeller soybean meal^[1](https://www.sciencedirect.com/science/article/pii/S0022030217306689?via%3Dihub#tbl1fn1)^ | — | 5.80 |
| Protein supplement^[2](https://www.sciencedirect.com/science/article/pii/S0022030217306689?via%3Dihub#tbl1fn2)^ | — | 0.78 |
| Urea | 0.46 | 0.59 |
| Soychlor^[3](https://www.sciencedirect.com/science/article/pii/S0022030217306689?via%3Dihub#tbl1fn3)^ | — | 1.23 |
| Saturated fat supplement^[4](https://www.sciencedirect.com/science/article/pii/S0022030217306689?via%3Dihub#tbl1fn4)^ | — | — |
| Limestone | — | — |
| Salt | 0.40 | — |
| Dicalcium phosphate | 0.50 | 0.52 |
| Magnesium oxide | — | — |
| Magnesium sulfate | 1.90 | 2.08 |
| Sodium bicarbonate | — | — |
| Mineral vitamin mix^[5](https://www.sciencedirect.com/science/article/pii/S0022030217306689?via%3Dihub#tbl1fn5)^ | 0.40 | 0.17 |
| Vitamin A^[6](https://www.sciencedirect.com/science/article/pii/S0022030217306689?via%3Dihub#tbl1fn6)^ | — | 0.03 |
| Vitamin D^[7](https://www.sciencedirect.com/science/article/pii/S0022030217306689?via%3Dihub#tbl1fn7)^ | — | 0.03 |
| Vitamin E^[8](https://www.sciencedirect.com/science/article/pii/S0022030217306689?via%3Dihub#tbl1fn8)^ | 0.40 | 0.60 |
| Biotin^[9](https://www.sciencedirect.com/science/article/pii/S0022030217306689?via%3Dihub#tbl1fn9)^ | — | 0.70 |
| Momensin^[10](https://www.sciencedirect.com/science/article/pii/S0022030217306689?via%3Dihub#tbl1fn10)^ | 0.01 | — |
| Ethyl-cellulose RPM^[11](https://www.sciencedirect.com/science/article/pii/S0022030217306689?via%3Dihub#tbl1fn11)^ | — | 0.09 |

^1^SoyPlus, West Central Soy (Ralston, IA, USA).

^2^ProVAAl AADvantage, Perdue AgriBusiness (Salisbury, MD, USA).

^3^West Central Soy.

^4^Energy Booster 100, Milk Specialties Global (Eden Prairie, MN, USA).

^5^Contained a minimum of 5% Mg, 10% S, 7.5% K, 2.0% Fe, 3.0% Zn, 3.0% Mn, 5,000 mg of Cu/kg, 250 mg of I/kg, 40 mg of Co/kg, 150 mg of Se/kg, 2,200 kIU of vitamin A/kg, 660 kIU of vitamin D_3_/kg, and 7,700 IU of vitamin E/kg.

^6^Contained 30,000 kIU/kg.

^7^Contained 5,000 kIU/kg.

^8^Contained 44,000 kIU/kg.

^9^ADM Animal Nutrition (Quincy, IL, USA).

^10^Rumensin, Elanco Animal Health (Greenfield, IN, USA).

^11^Ethyl-cellulose rumen-protected methionine, Evonik Nutrition and Care GmbH (Hanau-Wolfgang, Germany). Added only in the treatment group.

**TABLE S2.** Nutrient composition (mean ± standard deviation) and diet evaluation using NRC (2001) of maternal diets fed to multiparous Holstein cows prepartum.

| **Item** | **Far-off** | **Close-up** | |
| --- | --- | --- | --- |
|  |  | **CON** | **MET** |
| Chemical composition, % DM |  |  |  |
| CP | 13.9 ± 0.25 | 15.6 ± 0.32 | 15.7 ± 0.32 |
| NDF | 54.5 ± 0.75 | 40.8 ± 0.68 | 40.7 ± 0.68 |
| ADF | 36.9 ± 0.65 | 27.5 ± 0.50 | 27.4 ± 0.50 |
| NFC | 24.7 ± 0.72 | 34.9 ± 0.81 | 34.9 ± 0.81 |
| Ether extract | 1.81 ± 0.04 | 2.32 ± 0.05 | 2.33 ± 0.05 |
| Calculated using NRC (2001)[1](https://www.sciencedirect.com/science/article/pii/S0022030217306689?via%3Dihub#tbl2fn1) |  |  |  |
| NE_L_, Mcal/kg of DM | 1.33 | 1.47 | 1.47 |
| RDP, % of DM^2^ | 8.8 | 9.4 | 9.4 |
| RUP, % of DM^3^ | 5.1 | 6.2 | 6.3 |
| RDP required, g/d | 1,149 | 1,194 | 1,196 |
| RDP supplied, g/d | 1,157 | 1,204 | 1,203 |
| RDP balance, g/d | 8 | 10 | 7 |
| RUP required, g/d | 131 | 119 | 130 |
| RUP supplied, g/d | 668 | 793 | 924 |
| RUP balance, g/d | 537 | 673 | 794 |
| MP required, g/d^4^ | 790 | 808 | 808 |
| MP supplied, g/d | 1,211 | 1,363 | 1,473 |
| MP balance, g/d | 421 | 555 | 664 |
| Lysine , % of MP | 6.74 | 6.54 | 6.51 |
| MP-Lysine, g | 82 | 89 | 89 |
| Methionine, % of MP | 1.77 | 1.73 | 2.30 |
| MP-Methionine, g | 21 | 24 | 32 |
| Lysine:Methionine | 3.81:1 | 3.71:1 | 2.81:1 |

^1^The NRC (2001) evaluation of diets was based on final averaged prepartum DMI, production data, and feed analysis.

^2^Rumen degradable protein

^3^Rumen undegradable protein

^4^Metabolozable protein

**TABLE S3.** Number of 16S rRNA amplicon sequences (± standard deviation) in feces at birth and during the preweaning period in heifer calves born to cows offered a control diet (CON, n = 13) supplemented with ethyl-cellulose rumen-protected methionine (MET, n = 13; Mepron^®^ at 0.09% of diet DM; Evonik Nutrition & Care GmbH, Germany) compared with heifer calves born to cows offered a control diet (CON, n = 13) during the last 28 d of pregnancy

| **Day** | **CON** | **MET** |
| --- | --- | --- |
| 0 | 58,460 ± 19,589 | 58,117 ± 24,864 |
| 14 | 59,304 ± 18,198 | 57,384 ± 14,636 |
| 28 | 47,300 ± 17,237 | 45,955 ± 10,961 |
| 42 | 57,599 ± 18,513 | 56,120 ± 11,354 |
